# Role of hypothetical protein PA1-LRP in antibacterial activity of endolysin from a new *Pantoea* phage PA1

**DOI:** 10.3389/fmicb.2024.1463192

**Published:** 2024-10-23

**Authors:** Ye Tian, Xinyan Xu, Munazza Ijaz, Ying Shen, Muhammad Shafiq Shahid, Temoor Ahmed, Hayssam M. Ali, Chengqi Yan, Chunyan Gu, Jianfei Lu, Yanli Wang, Gabrijel Ondrasek, Bin Li

**Affiliations:** ^1^State Key Laboratory of Rice Biology and Breeding, Ministry of Agriculture Key Laboratory of Molecular Biology of Crop Pathogens and Insects, Zhejiang Key Laboratory of Biology and Ecological Regulation of Crop Pathogens and Insects, Institute of Biotechnology, Zhejiang University, Hangzhou, China; ^2^Station for the Plant Protection & Quarantine and Control of Agrochemicals of Zhejiang Province, Hangzhou, China; ^3^Department of Plant Sciences, College of Agricultural and Marine Sciences, Sultan Qaboos University, Al-Khoud, Muscat, Oman; ^4^Xianghu Laboratory, Hangzhou, China; ^5^Department of Life Sciences, Western Caspian University, Baku, Azerbaijan; ^6^Department of Botany and Microbiology, College of Science, King Saud University, Riyadh, Saudi Arabia; ^7^Crop Institute, Ningbo Academy of Agricultural Sciences, Ningbo, China; ^8^Institute of Plant Protection and Agricultural Product Quality and Safety, Anhui Academy of Agricultural Sciences, Hefei, China; ^9^State Key Laboratory for Managing Biotic and Chemical Threats to the Quality and Safety of Agro-Products, Institute of Plant Protection and Microbiology, Academy of Agricultural Sciences, Zhejiang, Hangzhou, China; ^10^Faculty of Agriculture, University of Zagreb, Svetošimunska Cesta, Zagreb, Croatia

**Keywords:** phage, endolysin, lysis, novel lysed protein, fusion expression

## Abstract

**Introduction:**

*Pantoea ananatis* has emerged as a significant plant pathogen affecting various crops worldwide, causing substantial economic losses. Bacteriophages and their endolysins offer promising alternatives for controlling bacterial infections, addressing the growing concerns of antibiotic resistance.

**Methods:**

This study isolated and characterized the *Pantoea* phage PA1 and investigated the role of PA1-LRP in directly damaging bacteria and assisting endolysin PA1-Lys in cell lysis, comparing its effect to exogenous transmembrane domains following the identification and analysis of the PA1-Lys and the PA1-LRP based on whole genome analysis of phage PA1. Additionally, this study also explored how hydrophobic region of PA1-LRP (HPP) contributes to bacterial killing when combined with PA1-Lys and examined the stability and lytic spectrum of PA1-Lys under various conditions.

**Results and discussion:**

Phage PA1 belonging to the *Chaseviridae* family exhibited a broad host range against *P. ananatis* strains, with a latent period of 40 minutes and a burst size of 17.17 phages per infected cell. PA1-Lys remained stable at pH 6-10 and temperatures of 20-50°C and showed lytic activity against various Gram-negative bacteria, while PA1-Lys alone could not directly lyse bacteria, its lytic activity was enhanced in the presence of EDTA. Surprisingly, PA1-LRP inhibited bacterial growth when expressed alone. After 24 h of incubation, the OD_600_ value of pET28a-LRP decreased by 0.164 compared to pET28a. Furthermore, the lytic effect of co-expressed PA1-LRP and PA1-Lys was significantly stronger than each separately. After 24 h of incubation, compared to pET28a-LRP, the OD_600_ value of pET28a-Lys-LRP decreased by 0.444, while the OD_420_ value increased by 3.121. Live/dead cell staining, and flow cytometry experiments showed that the fusion expression of PA1-LRP and PA1-Lys resulted in 41.29% cell death, with bacterial morphology changing from rod-shaped to filamentous. Notably, PA1-LRP provided stronger support for endolysin-mediated cell lysis than exogenous transmembrane domains. Additionally, our results demonstrated that the HPP fused with PA1-Lys, led to 40.60% cell death, with bacteria changing from rod-shaped to spherical and exhibiting vacuolation. Taken together, this study provides insights into the lysis mechanisms of *Pantoea* phages and identifies a novel lysis-related protein, PA1-LRP, which could have potential applications in phage therapy and bacterial disease control.

## 1 Introduction

In recent years, *Pantoea ananatis* has emerged as a significant rice pathogen across numerous countries, causing a spectrum of detrimental effects including grain discoloration, reduced seed germination rates, tissue decay, and leaf sheath necrosis, ultimately resulting in substantial yield losses (Xue et al., 2021; Zhang et al., 2022). Additionally, *P. ananatis* can lead to reduced production of wheat, corn, mulberry, strawberry, and water bamboo, and even cause plant death (Krawczyk et al., 2020; Wu et al., 2021; Xiao et al., 2022; Yuan et al., 2023). As natural enemies of bacteria, phages can specifically identify and effectively kill target bacteria, and they can co-evolve with host bacteria, thus exhibiting excellent biological safety (Piel et al., 2022; Rehman et al., 2019). In agriculture, phages are considered the most promising biopesticides (Ahmed and Li, 2023; Jamal et al., 2019). Phages have strong environmental adaptability, making them widely distributed in various environments, even extreme ones (Chevallereau et al., 2022; Hatfull et al., 2022). However, their high host specificity and bacterial resistance limit the application of phages (Luong et al., 2020).

Endolysins, encoded by phages and synthesized by host bacteria, are classified based on their functions as lytic transglycosidases, lysozyme, amidases, glycosidases and endopeptidases (Wong et al., 2022). Compared to phages, endolysins with lower host specificity and higher lethality have recently been explored as antibacterial agents (Ho et al., 2022; Lai et al., 2020). In addition to their individual antibacterial effectiveness, endolysins can also synergistically act with antibiotics, showing broad application prospects in the prevention and treatment of multidrug-resistant pathogen infections (Ghose and Euler, 2020; Hong et al., 2022). In the future, endolysins have broad application prospects in many industries such as food, feed, detergents, and pharmaceuticals (Cho et al., 2021; Pallesen et al., 2023; Shannon et al., 2020). Endolysin can directly hydrolyze the peptidoglycan cell wall of Gram-positive bacteria, causing cell lysis. However, the outer membrane of Gram-negative bacteria, which contains lipopolysaccharides, acts as a barrier preventing most endolysin from directly entering the cell. Outer membrane permeation agents such as chloroform and ethylenediaminetetraacetic acid (EDTA) have been reported to alter cell membrane permeability, thereby helping endolysins enter Gram-negative bacteria and inhibit their growth (Yuan et al., 2021).

The holin-endolysin pathway is the most common phage lysis mechanism for phages of Gram-negative bacteria (Basit et al., 2021). Holins are small hydrophobic proteins with at least one transmembrane domain (TMD), forming holes in the inner membrane to help endolysins hydrolyze peptidoglycan, ultimately leading to cell death (Brüser and Mehner-Breitfeld, 2022). Additionally, some phages produce endolysins that can traverse the plasma membrane without the assistance of holins. In the phages of Gram-negative bacteria, some endolysins contain a signal-anchor-release (SAR) domain at their N-terminal, allowing them to autonomously split cells (Cahill and Young, 2019). Besides endolysin and holin operation, membrane fusion proteins are required for bacterial lysis (Gontijo et al., 2021). The direct fusion of endolysins and antimicrobial peptides not only enhances antibacterial activity, but also expands the antibacterial spectrum (Gouveia et al., 2022). Furthermore, endolysins modified with cationic/hydrophobic amino acids significantly enhance bacterial cell membrane permeability and effectively kill bacteria (Wang et al., 2017).

In this study, we isolated and characterized the *Pantoea* phage PA1. We identify and analyze the endolysin (PA1-Lys) and the hypothetical protein PA1-LRP based on whole genome analysis of phage PA1. Additionally, we investigated the role of PA1-LRP in directly damaging bacteria and assisting PA1-Lys in cell lysis, comparing its effect to exogenous TMD. We also explored how hydrophobic region of PA1-LRP (HPP) contributes to bacterial killing when combined with PA1-Lys. Furthermore, we examined the stability and lytic spectrum of PA1-Lys under various conditions. Overall, our study provides a theoretical basis for understanding the lysis mechanisms of *Pantoea* phages and potentially develop new strategies for bacterial control.

## 2 Materials and methods

### 2.1 Bacterial strains, plasmid, and reagents

In this study, strains of *P. ananatis* (ZJU1 - ZJU14), *Escherichia coli* (DH5α, BL21 (DE3), S17, and XL1-Blue), *Xanthomonas oryzae* pv. *oryzae* (Xoo) (Y4, PXO99^A^, and C2), *Xanthomonas oryzae* pv. *oryzicola* (Xoc) (BLS256 and RS105), *Acidovorax avenae* subsp. *avenae* (Ao) (RS1 and RS2), *Pantoea dispersa* strain 19,001, *Bacillus* sp. strain RP12, and *Paenibacillus polymyxa* strain RP31 stored in our laboratory were utilized. *E. coli* strains were cultured in Luria-Bertani (LB) broth at 37°C, while other strains were cultured in nutrient broth (NB) at 30°C. The *E. coli* BL21 strains containing plasmids pET28a, pET28a-Lys, pET28a-LRP, pET28a-Lys-LPP, pET28a-Lys-LRP, pET28a-Lys-LVP, pET28a-Lys-ATMD, and pET28a-Lys-HPP were abbreviated as 28a, 28a-Lys, 28a-LRP, 28a-Lys-LPP, 28a-Lys-LRP, 28a-Lys-LVP, 28a-Lys-ATMD, and 28a-Lys-HPP, respectively. 28a and 28a-Lys were used as negative controls to study the lytic activity of the lytic related proteins (Table 1).

**Table 1:** Plasmids used in this study.

Plasmids	Description	Sources
pET28a	Km^R^; cloning vector	Laboratory collection
pBT	Chl^R^; encode λcI; bait plasmid	Laboratory collection
pTRG	Tet^R^; encode α-NTD; target plasmid	Laboratory collection
pET28a-Lys	Km^R^; pET28a containing gene Lys	This study
pET28a-LRP	Km^R^; pET28a containing gene LRP	This study
pET28a-Lys-LPP	Km^R^; pET28a containing gene Lys and LPP	This study
pET28a-Lys-LRP	Km^R^; pET28a containing gene Lys and LRP	This study
pET28a-Lys-LVP	Km^R^; pET28a containing gene Lys and LVP	This study
pET28a-Lys-ATMD	Km^R^; pET28a containing gene Lys and ATMD	This study
pET28a-Lys-HPP	Km^R^; pET28a containing gene Lys and HPP	This study
pBT-Lys	Chl^R^; pBT containing gene Lys	This study
pTRG-LRP	Tet^R^; pTRG containing gene LRP	This study

Km^R^/Chl^R^/Tet^R^: kanamycin−/ chloramphenicol−/ tetracycline-resistant.

Moreover, the concentrations of antibiotics we used in this study were as follows: kanamycin at 50 μg/mL, tetracycline at 12.5 μg/mL, or chloramphenicol at 34 μg/mL. SM buffer (pH 7.5) consists of 8 mM magnesium sulfate, 0.1 mM sodium chloride, 1% gelatin and 50 mM Tris–HCl, which was used for preservation and dilution of phages. Furthermore, Isopropyl-*β*-D-thiogalactopyranoside (IPTG) was used as a protein inducer at a final concentration of 0.5 mmol/L. In addition to this, the Live/Dead Bacterial Staining Kit was purchased from Thermo Fisher Scientific (Waltham, MA, USA). Lysozyme used as a positive control is a commercial lysozyme extracted from egg whites, which was purchased from Beyotime Biotechnology (Haimen, China). Besides, the BCA protein quantitative kit, Phosphate Buffer Solution (PBS), EDTA, Tris–HCl buffer and IPTG were also purchased from Beyotime Biotechnology (Haimen, China).

### 2.2 Isolation, purification, and morphology of phage PA1

To isolate phages, we utilized a traditional isolation method devised by Liu et al. (2021). Rice leaves were ground using a sterilized mortar, and the resulting homogenate was centrifuged at 11,000 × g for 10 min to obtain the supernatant. The supernatant was then filtered through a 0.22 μm filter (Millipore, Ireland) to remove bacteria completely. Various *P. ananatis* strains (ZJU1-ZJU14) were selected to identify the presence of phages in the supernatant. The supernatant filtrate was spotted onto double-layer plates containing 1 mL of each *P. ananatis* suspension and 7 mL of molten NB semi-solid broth (0.8% agar). After air drying, the plates were incubated overnight at 30°C. The presence of transparent plaques on the double-layer plates indicated the presence of lytic phages. These plaques were picked with sterile tips and placed in SM buffer, and the mixed SM buffer was left overnight at 4°C. After that, 7 mL of molten NB semi-solid broth, 1 mL of *P. ananatis* ZJU1, and 100 μL of the diluted phage SM buffer were mixed and poured onto NB plates. A single plaque was then selected and the process was repeated five times until a uniform single transparent plaque appeared. At this point, the purified phage was named PA1. For higher titers of phage samples, the purified phage was concentrated according to previous studies (Sasaki et al., 2021). Finally, for morphological and structural observation, the PA1 phage suspension was placed on carbon-coated copper grids (Ted Pella Inc., USA) and observed under a transmission electron microscope (TEM) (JEM-1230, JEOL, Akishima, Japan).

### 2.3 Phage host range and optimal multiplicity of infection (MOI)

The spot assay method, as described by Huang et al. (2022), was used to investigate the host range of phage PA1. In this method, a 5 μL suspension of phage PA1 was applied onto double-layer agar plates containing lawns of various bacterial strains, followed by incubation at 30°C. The presence of clear, transparent spots signified bacterial susceptibility to phage PA1. To determine the optimal multiplicity of infection (MOI), we adapted and modified the protocol outlined in Cao et al. (2022). Given the high lytic efficiency of phage PA1 against *P. ananatis* ZJU1, this particular strain was selected for further investigation. During the exponential growth phase of *P. ananatis* ZJU1, different ratios of phage PA1 to *P. ananatis* ZJU1 (ranging from 1:1000 to 1,000:1) were mixed in NB and incubated at 30°C for 4 h. Post-incubation, the mixtures were centrifuged at 12,000 × g for 10 min and subsequently filtered to isolate the supernatant. The phage titer was then quantified using the double-layer plate method, with the MOI yielding the highest phage titer identified as optimal. This experiment was conducted in triplicate, with each replication performed independently.

### 2.4 Phage adsorption rate and one-step growth curve

The phage adsorption rate was examined following the methodology devised by Xuan et al. (2022). Briefly, *P. ananatis* ZJU1 at a concentration of 1 × 10^6^ CFU/mL was infected with phage PA1 at the predetermined optimal MOI of 0.01. Supernatant samples were collected at multiple time points: 0, 1, 2, 3, 4, 5, 6, 7, 8, 9, 10, 15, 20, 25, and 30 min. The quantity of unabsorbed phage particles was determined using the double-layer plate assay. The adsorption rate was calculated using the formula: adsorption rate (%) = [(initial phage titer – unabsorbed phage titer)/initial phage titer] × 100%. The one-step growth assay was conducted in accordance with the protocol established by Gašić et al. (2022). Specifically, once *P. ananatis* ZJU1 reached its exponential phase, phage PA1 was introduced at an MOI of 0.01. The mixture was incubated at 30°C for 20 min, followed by centrifugation at 12,000 × g for 2 min at 4°C to remove unabsorbed phages, after which the supernatant was discarded. The resultant pellet was resuspended in NB broth and incubated at 30°C. Samples were taken at 20-min intervals up to 240 min. The supernatant samples were then filtered and subjected to gradient dilution. The phage titer was subsequently assessed via the double-layer plate method. All experiments were performed in triplicate to ensure reproducibility.

### 2.5 Phage stability

To elucidate the factors influencing the stability of phage PA1, the double-layer plate method was used to ascertain the phage titer following various treatments. Drawing upon previous studies, both temperature and pH stability assays were performed. The pH stability experiment involved incubating phage PA1 in SM buffer across a pH gradient ranging from 3 to 12 at 25°C for 60 min. For the thermal resistance assay, phage PA1 was exposed to a series of temperatures (4, 25, 40, 50, 60, 70, and 80°C) at a neutral pH of 7 for a duration of 1 h. The activity of the treatment exhibiting the highest phage titer was designated as 100%. Each experiment was conducted in triplicate, with all replicates executed independently to ensure robustness and reproducibility of the results.

### 2.6 Genome sequencing and phylogenetic analysis of phage PA1

Genomic DNA of phage PA1 was extracted utilizing the *λ* phage genome kit (Sangon Biotech, Shanghai, China). Whole genome sequencing was subsequently performed at Biozeron (Shanghai, China) employing the Illumina HiSeq paired-end platform, with quality control of the raw reads conducted using Trimmomatic. Following trimming, the paired-end reads were assembled via AbySS.^1^ Open reading frames (ORFs) were predicted using both the RAST server,^2^ GeneMarkS^3^ and Pharokka.^4^ Protein functions were annotated within the non-redundant protein database through BLASTp.^5^ The detection of tRNA was executed using tRNAscan-SE v2.0.^6^ A circular representation of the phage PA1 genome was generated using Proksee^7^ as outlined by Vu et al. (2021). Phylogenetic trees, based on the phage PA1 genome and TERL (termination enzyme large subunit), were constructed using MEGA6 software.

### 2.7 Recombinant plasmid construction

We have named the proteins encoded by ORF11, ORF12, ORF13, and ORF16 as PA1-LPP, PA1-Lys, PA1-LRP, and PA1-LVP, respectively. In order to construct recombinant plasmids, the genes encoding PA1-Lys, PA1-LPP, PA1-LRP, and PA1-LVP were amplified using phage PA1 as a template, and the gene encoding ATMD was amplified using Ao phage AP1 as a template. Cloning of various gene fragments into different plasmids were done using specific restriction enzymes and T4 ligases. The conjugated product was transfected into *E. coli* DH5α by the heat shock method to obtain a recombinant plasmid. Gene sequences used in this study are listed Supplementary Table S2 and the primers used in this study are listed in Table 2.

**Table 2:** PCR primers used in this study.

Primer Name	Nucleotide Sequence (5′–3′)	Characterization
28a-Lys-F	GGAATTCCATATGATGATCAGTAAAAACGCAATTG (N)	Gene of PA1-Lys from phage PA1
28a-Lys-R	CGGGATCCTAAGAATCTCAAGCAATCTTTATAACG (B)	
28a-LRP-F	CGGGATCCATGGTAAGGCGCAGACG (B)	Gene of PA1-LRP from phage PA1
28a-LRP-R	CCGCTCGAGCTTTTGCTGTTCTACTTTCTGGA (X)	
28a-LPP-F	CGGGATCCATGGTTCAAATCTACGTCAGGC (B)	Gene of PA1-LPP from phage PA1
28a-LPP-R	CCGCTCGAGACTAGAACGGAACCAGCCG (X)	
28a-LVP-F	CGGGATCCATGAACTGGCAAGACATTGG (B)	Gene of PA1-LVP from phage PA1
28a-LVP-R	CCGCTCGAGTAACGGTAGACCTAACTCAGC (X)	
ATMD-F	CGGGATCCAGCCTCGGCAACTGGC (B)	Gene of ATMD
ATMD-R	CCGCTCGAGTGACCACCCCTCTCGCC (X)	
HPP-F	CGGGATCCAGGACTATTTTGATCAGCTG (B)	Gene of HPP
HPP-R	CCGCTCGAGAGCAGGCGCTAAGTCATAG (X)	
PBT-Lys-F	CGGAATTCATGATCAGTAAAAACGCAATTG (E)	Gene of PA1-Lys from phage PA1
PBT-Lys-R	CGGGATCCTTATAAGAATCTCAAGCAATCTTTATAACG (B)	
PTRG-LRP-F	ATAAGAATGCGGCCGCATGGTAAGGCGCAGACG (No)	Gene of PA1-LRP from phage PA1
PTRG-LRP-R	CGGAATTCTTACTTTTGCTGTTCTACTTTCTGGA (E)	

Lys, PA1-Lys; LRP, PA1-LRP; LPP, PA1-LPP; LVP, PA1-LVP; ATMD, transmembrane domain of endolysin in phage AP1; HPP, hydrophobic region of PA1-LRP. The parentheses contain the abbreviation for restriction endonuclease and the cleavage site is marked with an underline (N, NdeI; B, BamHI; X, XhoI; E, EcoRI; No, NotI).

### 2.8 Bioinformatics and physicochemical properties analysis

ExPASY prot param tool^8^ was used to predict the basic physicochemical properties of PA1-Lys, including isoelectric point, stability index, aliphatic index, and hydrophobicity. Analysis of protein conserved functional domains was obtained through the Conserved Domain Database in NCBI.^9^ Application of TMHMM-2.0^10^ and SignalP-5.0 Server^11^ to analyze the TMD and signal peptide. SWISS-MODEL^12^ and phyre2 analysis^13^ were used to predict the structure of protein.

### 2.9 Expression and purification of PA1-Lys

The recombinant *E. coli* 28a-Lys was cultured overnight in LB broth containing 50 μg/mL kanamycin at 37°C and 200 rpm. Following this, 200 μL of the overnight culture was inoculated into 200 mL of LB broth and incubated at 37°C. Upon reaching an OD_600_ of 0.6, protein expression was induced by adding 200 μL of 0.5 M IPTG, followed by incubation at 20°C for 20 h. The bacterial cells were harvested by centrifugation, washed three times with 0.1 M PBS, and resuspended in 0.1 M PBS. The cells were then lysed using an ultrasonic homogenizer (400 W, 5 s on, 10 s off) for a total of 10 min. The resulting lysate was centrifuged at 11,000 × g for 30 min at 4°C to separate the supernatant and sediment. Protein purification was performed using the ProteinIso™ Ni-NTA Resin (Genscript, China). After elution with imidazole, the purified protein was analyzed by SDS-PAGE and Western blotting. The PA1-Lys protein was concentrated using a 3 kDa protein ultrafiltration tube (Pell, USA), and the concentrated PA1-Lys was stored in 50 mM Tris–HCl buffer (pH 8.0). Protein concentration was determined using the BCA protein quantitation kit.

### 2.10 Lytic capacity of PA1-Lys

#### 2.10.1 Turbidity reduction assay

The turbidity reduction assay was conducted with slight modifications to the previously described method by Zhang et al. (2021). Overnight cultures of *P. ananatis* strain ZJU1 were centrifuged, and the resulting bacterial pellet was resuspended in 0.1 M PBS and then treated with 0.5% (*v/v*) chloroform for 5 min. The treated bacteria were centrifuged at 6,000 × g for 4 min, and the resulting pellet was washed three times with 0.1 M PBS. The bacterial pellet was subsequently resuspended in 50 mM Tris–HCl buffer (pH 8.0) containing 0.1% Triton X-100. The 20 μL PA1-Lys (0.2 mg/mL) was added to 200 μL of chloroform-treated bacterial suspension, and the value of OD_450_ was measured every 5 min by a microplate photometer (Thermo Fisher Scientific Inc., Waltham, MA, USA). Lysozyme (0.2 mg/mL) was utilized as a positive control, while 0.1 M PBS buffer served as a negative control. Each experiment was repeated independently three times to ensure reproducibility.

#### 2.10.2 Synergistic lysis of viable Bacteria by PA1-Lys and EDTA

Overnight cultures of *P. ananatis* strain ZJU1 were centrifuged, and the resulting bacterial pellet was resuspended in 50 mM Tris–HCl buffer (pH 8.0) to achieve a suspension with an OD_600_ of 0.8 (equivalent to 2 × 10^8^ CFU/mL). To this bacterial suspension, a mixture comprising 100 μL of recombinant PA1-Lys protein (0.2 mg/mL) and 100 μL of EDTA solution (0.01 mol/L) was added to 800 μL of the bacterial suspension. As a control, 100 μL of 50 mM Tris–HCl buffer (pH 8.0) was combined with 100 μL of PA1-Lys and 800 μL of the bacterial suspension. Following incubation at 25°C for both 30 and 60 min, bacterial counts were determined using plate counting methods. Each experiment was independently repeated three times to ensure the validity and reproducibility of the results.

#### 2.10.3 Lysis spectrum of PA1-Lys

*Pantoea ananatis*, *P. dispersa*, *E. coli*, Xoo, Xoc, Ao, *P. polymyxa* and *Bacillus* were used as indicator bacteria to determine the lytic spectrum of PA1-Lys. Gram-negative bacteria require pre-treatment with chloroform, while Gram-negative bacteria do not require treatment. The lytic effect of PA1-Lys was measured using a turbidity analysis method.

$\mathrm{Lytic activity}\;\left(\%\right)=\left[\begin{array}{l} {(OD}_{450}\;\mathrm{value of bacteria before}\;PA1-Lys\;\mathrm{treatment)}\\ –\;{OD}_{450}\mathrm{value}\;\mathrm{of}\;\mathrm{bacteria}\;\mathrm{after}\;PA1-\;Lys\;\mathrm{treatment}\\ /{OD}_{450}\;\mathrm{value of bacteria before}\;PA1-Lys\;\mathrm{treatment} \end{array}\right]\times 100\%\text{.}$

### 2.11 Biochemical characterization of PA1-Lys

The method described by Jiang et al. (2021) was used to determine the optimal conditions for PA1-Lys activity. To assess the thermal stability of PA1-Lys, the protein was subjected to various temperatures (ranging from 20°C to 70°C) for durations of 30 and 60 min, respectively. The lytic activity was then evaluated at 25°C using a turbidity reduction assay. The effect of pH on the lytic activity was also investigated. Specifically, overnight cultures of *P. ananatis* strain ZJU1 were centrifuged at 6,000 × g for 4 min, and the resulting bacterial pellet was resuspended in 0.1 M PBS. A mixture of 20 μL PA1-Lys and 200 μL chloroform was added to the bacterial suspension. The pH of the suspension was adjusted to a range from 2 to 11 using sodium hydroxide or hydrochloric acid. The OD_450_ value was monitored after 30 and 60 min of incubation at 25°C. Additionally, the influence of various metal ions on lytic activity was assessed. Metal ions such as FeCl_3_, ZnCl_2_, CuCl_2_, MnCl_2_, NaCl, KCl, FeCl2, MgCl_2_, and CaCl_2_ were added to the chloroform-treated bacterial suspension at a final concentration of 10 mmol/L each. Subsequently, 20 μL PA1-Lys was added to 200 μL of the treated bacterial resuspension. The OD_450_ value was then monitored every 5 min at 25°C. Each experiment was independently repeated three times to ensure the robustness and reproducibility of the results.

### 2.12 Bacterial growth assays

According to previous research (Wu et al., 2021), when the OD_600_ of BL21 strains containing the recombinant plasmid reached 0.4, IPTG (0.5 M, 5 μL) was added to 5 mL of bacterial culture. The induced bacteria were then cultured at 20°C and 180 rpm. At intervals of 6-, 12-, 18-, and 24-h post-induction, the absorbance at 600 nm of the BL21 strains was measured using a microplate reader (Thermo Fisher Scientific Inc., Waltham, MA, USA). This experiment was conducted three times independently to ensure reliability and consistency of the results.

### 2.13 Determination of *β*-galactosidase activity

To evaluate the effect of recombinant proteins on the permeability of the cell membrane, we measured β-Galactosidase activity as described previously with minor modifications (Ning H. et al., 2021). Bacterial suspensions induced by IPTG were centrifuged (12,000 × *g*, 5 min), and the supernatant was collected. The 100 μL 20 mM O-nitrophenyl-β-D-galactopyranoside (ONPG) was added to 500 μL extracellular supernatant. After placing it at 45°C for 30 min, an equal volume of Na_2_CO_3_ (0.5 mM) was added to terminate the reaction. The activity of galactosidase was evaluated by measuring the OD_420_ of the samples with a microplate reader.

### 2.14 Live/dead cell and flow cytometry assay

Following the methodology outlined by Wu et al. (2021), 5 μL of 0.5 M IPTG was added to a 5 mL bacterial suspension for induction at 20°C over a 24-h period. Subsequently, bacterial sediment was harvested by centrifugation at 6000 × g for 3 min and washed three times with 0.1 M PBS. To assess bacterial viability, the BacLight bacterial viability kit (Thermo-Fisher Scientific, Waltham, MA, USA), containing SYTO 9 and propidium iodide (PI) dyes, was utilized. Bacterial samples were stained with a mixture of SYTO 9 and PI in the dark for 30 min, followed by centrifugation (6,000 × g, 3 min) to remove the staining solution. Stained bacteria were examined using an Olympus inverted confocal microscope (Leica-SP8, Heidelberg, Germany). For flow cytometry analysis, the preparation of bacterial samples mirrored the live/dead experiments. PI dye was specifically used to identify dead bacteria. Bacterial samples were incubated with PI in the dark for 30 min, followed by washing with 0.1 M PBS to remove excess dye, as per the protocol described by Daei et al. (2022). Stained bacteria were then analyzed using the FACSVerse cytometer (BD Biosciences, San Jose, CA, USA). This approach allowed for precise quantification of bacterial viability and death under the experimental conditions.

### 2.15 TEM observation

The changes in the bacterial microstructure induced by IPTG were studied by TEM (JEM-1230, JEOL, Akishima, Japan). Bacterial pellets were fixed overnight with 2.5% (*v/v*) glutaraldehyde, coated with agar and washed 3 times with 0.1 M PBS. Subsequently, bacterial blocks were treated with 1% (*w*/*v*) osmic acid for 1 h. After washing three times with PBS, the bacterial samples were dehydrated in different concentrations of alcohol (30–80%, *v*/*v*). Afterwards, the samples were dehydrated in different acetone solutions (90–100%, *v*/*v*). After embedding, infiltration, and sectioning, the bacterial samples were observed.

### 2.16 Bacterial two-hybrid assay

The bacterial two-hybrid assay method was adapted from the protocol described by Liu et al. (2017). Initially, the PBT-Lys vector and PTRG-LRP vector were co-transformed into *E. coli* XL1-Blue, and subsequent screening was conducted to verify the correct dual hybrid vectors. *E. coli* XL1-Blue strains containing PBT-LGF2 and PTRG-Gal11^p^ served as positive controls, while strains containing PBT and PTRG acted as negative controls. Subsequently, 3 μL of bacterial suspension from each transformation was inoculated onto non-selective medium plates supplemented with corresponding antibiotics, as well as onto M9 + His-Dropout double selective media plates containing antibiotics, 3 mM 3-AT (3-amino-1, 2, 4-triazole), and 12.5 μg/mL streptomycin. All plates were then incubated at 25°C for 24 h. This experimental setup allowed for the selective growth and assessment of interactions between the proteins encoded by the co-transformed vectors under specific conditions.

## 3 Results

### 3.1 Biological characteristics of phage PA1

**Figure 1:**
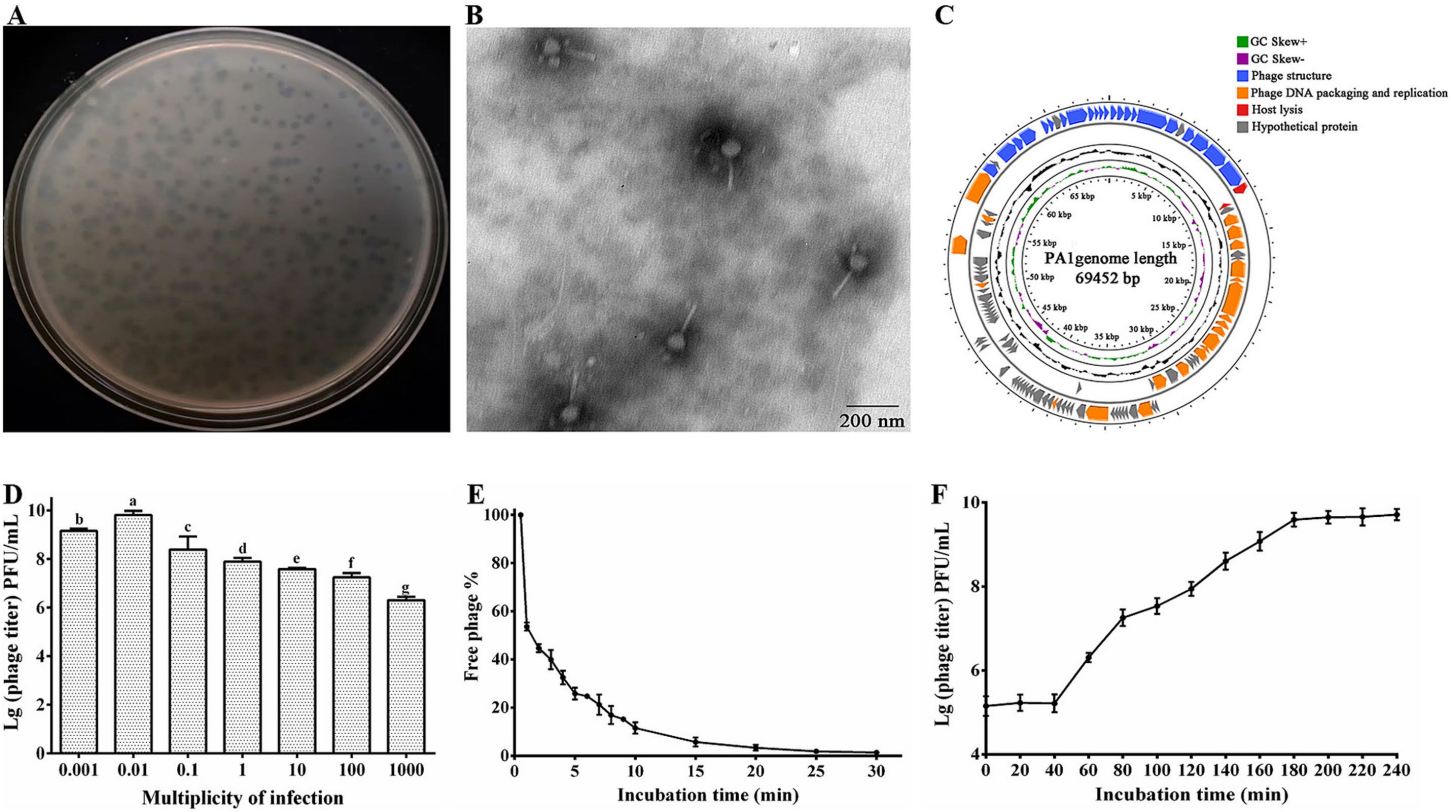
Morphological identification and biological features of phage PA1. **(A)** Plaque morphology of phage PA1. **(B)** TEM image of phage PA1 particles. Scale bar = 200 nm, Magnification = 12,000x. **(C)** Genome map of PA1. The open reading frames (ORFs) are indicated by specific colors according to their functional categories. The direction of each arrow represents the direction of transcription. Genes in four functional modules are represented in blue (phage structure), orange (phage DNA packaging and replication), red (host lysis), and gray (hypothetical protein). GC skew is shown as inner circles holograms in purple and green. GC content is indicated by black circular hologram. **(D)** Optimal multiplicity of infection (MOI) of phage PA1. **(E)** Adsorption assay of phage PA1. **(F)** One-step growth curve of phage PA1. Different letters on the Bar chart represents significant differences between samples. Error bars indicates the mean of three replicates (*n* = 3 ± standard deviation).

Our study characterized the isolated phage, which formed small, clear, circular plaques on *P. ananatis* ZJU1 (Figure 1A). Upon further purification, the isolated phage exhibited distinct features including bright patches, well-defined edges, and a uniform size (2 mm), leading to its designation as phage PA1. TEM analysis revealed that phage PA1 possesses a head diameter of 80 ± 6.2 nm and a contractile, flexible tail approximately 120 ± 5.4 nm in length (Figure 1B). According to the latest ICTV classification criteria, phage PA1 belongs to the *Chaseviridae* family. Sequencing of phage PA1’s genome unveiled a circular double-stranded DNA (dsDNA) with a length of 69,452 bp and an average G + C content of 46.1%. Using RAST servers, GeneMarkS and Pharokka, a total of 105 putative open reading frames (ORFs) were identified, with 46 located on the minus strand and the remainder on the plus strand. Among these, 41 ORFs were functionally annotated while 64 were categorized as hypothetical proteins. No transfer RNA (tRNA) sequences were detected within the phage genome using tRNAscan-SE.

Bioinformatics analysis revealed that the genome of phage PA1 is organized into four functional modules: phage structure, DNA packaging and replication, host lysis, and hypothetical proteins (Figure 1C). The genomic architecture of phage PA1 demonstrates a modular organization where genes with related functions are clustered together. The complete genome sequence of phage PA1 has been deposited in the NCBI database under accession number PP537389. This comprehensive genomic characterization provides insights into the genetic composition and functional potential of phage PA1 within its ecological niche.

Through bioinformatics prediction, we found that 22 genes related to phage structural function, including baseplate protein, tail fiber protein, head-tail connector protein, capsid protein, and tail sheath protein. Terminase large subunit encoded by ORF85 is responsible for phage genome packaging and phage protease encoded by ORF88 which plays a role in phage morphogenesis and is also present in DNA packaging module (Evseev et al., 2021; Golz and Kemper, 1999; Thomas et al., 2012). Replication-related genes encoding DNA polymerase, DNA helicase, DNA ligase, and other related proteins, bacterial lysis genes, and phage-encoded enzymes attacking peptidoglycans were studied in many previous studies (Vu et al., 2021; Yang et al., 2021; Young, 1992). PA1-Lys encoded by ORF12 is associated with host lysis. However, since most of the proteins encoded by the ORFs are predicted to be hypothetical proteins, further investigation of the roles of the encoded gene products is needed. In this study, it was discovered that the ORF13 encoded hypothetical protein (PA1-LRP) directly lyse bacteria. Hence, the PA1-LRP was classified into lysis module.

The research findings indicated that the optimal MOI for phage PA1 was determined to be 0.01 (Figure 1D). Adsorption experiments revealed that phage PA1 achieved an adsorption rate of 97% within 20 min (Figure 1E). To further elucidate the proliferation dynamics, including incubation time and burst size, a one-step growth curve was conducted. The curve illustrated that phage PA1 had a latent period of 40 min and a burst size of 17.17 phages per infected bacterial cell (Figure 1F). These results provided valuable insights into the infectivity and replication characteristics of phage PA1 under experimental conditions.

### 3.2 Biological characteristics of phage PA1

**Figure 2:**
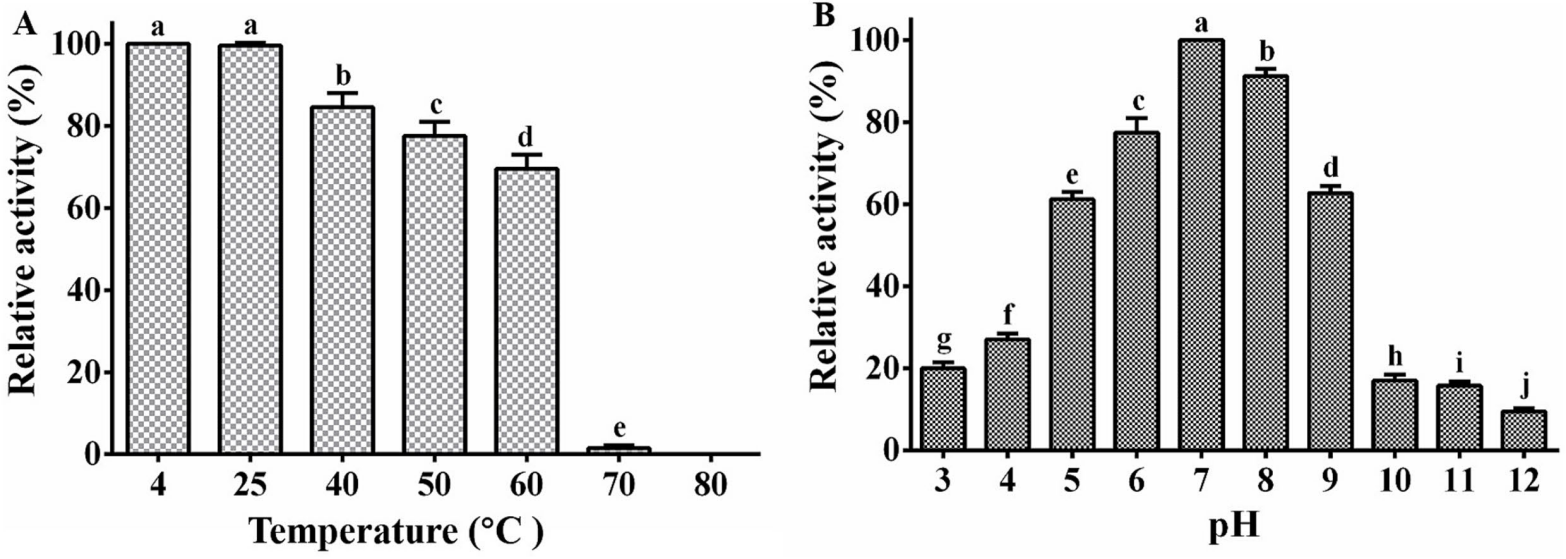
Stability of phage PA1. **(A)** Temperature stability of phage PA1. **(B)** pH stability of phage PA1. Different letters on the Bar chart represents significant differences between samples. Error bars indicates the mean of three replicates (*n* = 3 ± standard deviation).

Our study demonstrated that phage PA1 exhibited peak activity at 4°C and 25°C following treatment at various temperatures for 1 h. Phage activity declined sharply by 98.8% at 70°C and was completely deactivated at 80°C (Figure 2A), indicating high-temperature adaptability within a specific range. Additionally, phage PA1 exhibited optimal survival at pH 7, with a noticeable decrease in survival rates observed at pH 10 and pH 3 (Figure 2B). These results suggest that phage PA1 displays sensitivity to extreme acidic and alkaline conditions, highlighting its preference for neutral pH environments.

### 3.3 Host range of phage PA1

The host range test showed that phage PA1 has a broad host range, as it had lytic ability against all tested strains of *P. ananatis* (Table 3), but phage PA1 displayed different infection rates when tested against different strains of *P. ananatis*. Specifically, phage PA1 showed strong lytic ability against ZJU1, ZJU10 and ZJU13 strains, but its lytic ability was limited when tested against ZJU7 and ZJU8 strains. Notably, phage PA1 did not have lytic ability on other bacterial genera.

**Table 3:** Host range of phage PA1.

Strain	Infection	Strain	Infection
*P. ananatis* ZJU1	**+++**	*P. ananatis* ZJU13	**+++**
*P. ananatis* ZJU2	**++**	*P. ananatis* ZJU14	**++**
*P. ananatis* ZJU3	**++**	*P. dispersa* 19,001	**−**
*P. ananatis* ZJU4	**++**	Xoo PXO99^A^	**−**
*P. ananatis* ZJU5	**++**	Xoo C2	**−**
*P. ananatis* ZJU6	**++**	Xoc BLS256	**−**
*P. ananatis* ZJU7	**+**	Xoc RS105	**−**
*P. ananatis* ZJU8	**+**	Ao RS1	**−**
*P. ananatis* ZJU9	**++**	Ao RS2	**−**
*P. ananatis* ZJU10	**+++**	*Bacillus* sp. RP12	**−**
*P. ananatis* ZJU11	**++**	*P. polymyxa* RP31	**−**
*P. ananatis* ZJU12	**++**		

“+++” means dense patches appearing on the plate, but the background clearness; “++” means dense patches appearing on the plate, but the background is blurry; “+” means only a few patches on the plate; “–” no patche on the plate. All strains were isolated from rice leaves.

### 3.4 Phylogenetic analysis

**Figure 3:**
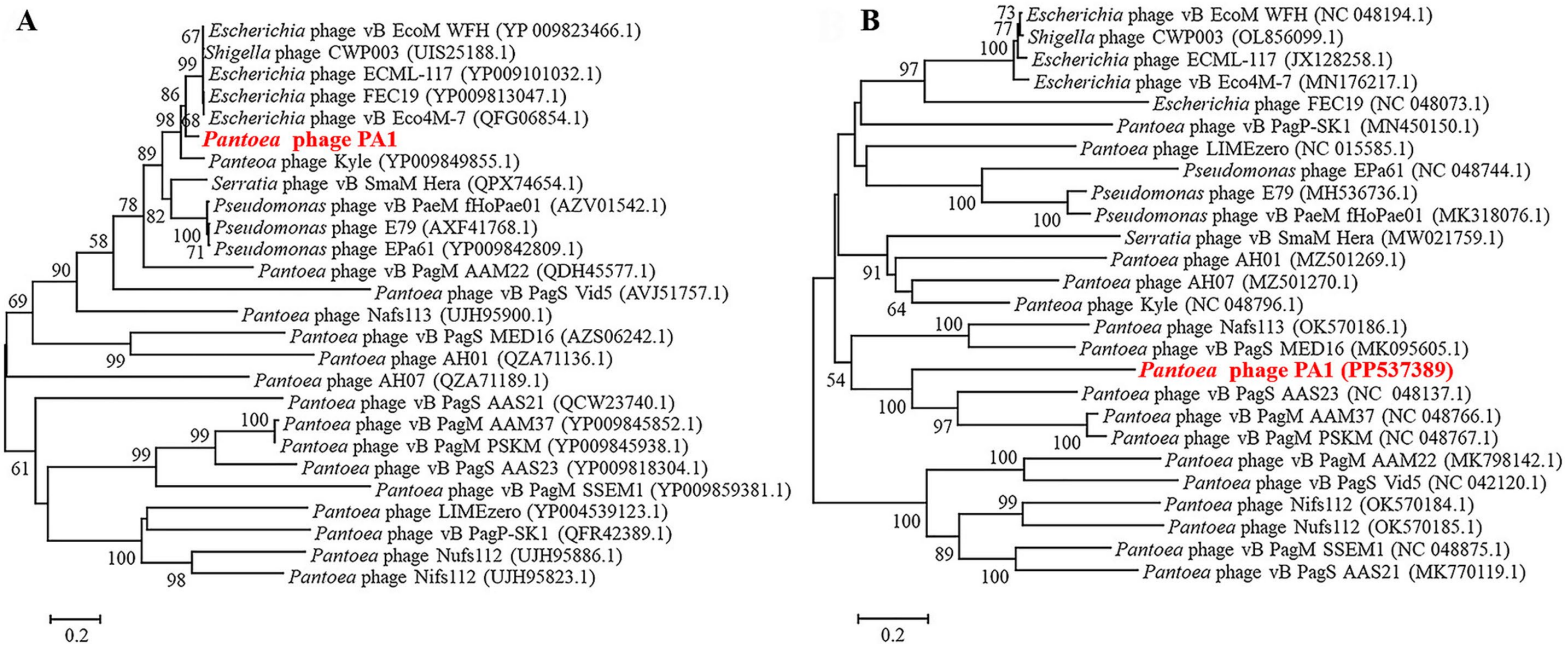
Neighbor-joining phylogenetic analysis of phage PA1. **(A)** Phylogenetic tree based on the amino acid sequences of terminal large subunit. **(B)** Phylogenetic tree based on Genome sequence.

Based on the phylogenetic analysis of the entire genome sequence and the amino acid sequence of the conserved protein (terminal large subunit), we found that phage PA1 clustered into a distinct branch. Phage PA1 is closely related to *Escherichia* phage based on the terminase large subunit (Figure 3A). However, according to the entire genome sequence, phage PA1 exhibits a phylogenetic relationship similar to that of *Pantoea* phages (Figure 3B). The whole genome sequencing of phage PA1 showed relatively low similarity to other phages in the NCBI database. We therefore believed that phage PA1 to be a new *Pantoea* phage.

### 3.5 Bioinformatics and physicochemical properties of PA1-Lys

**Figure 4:**
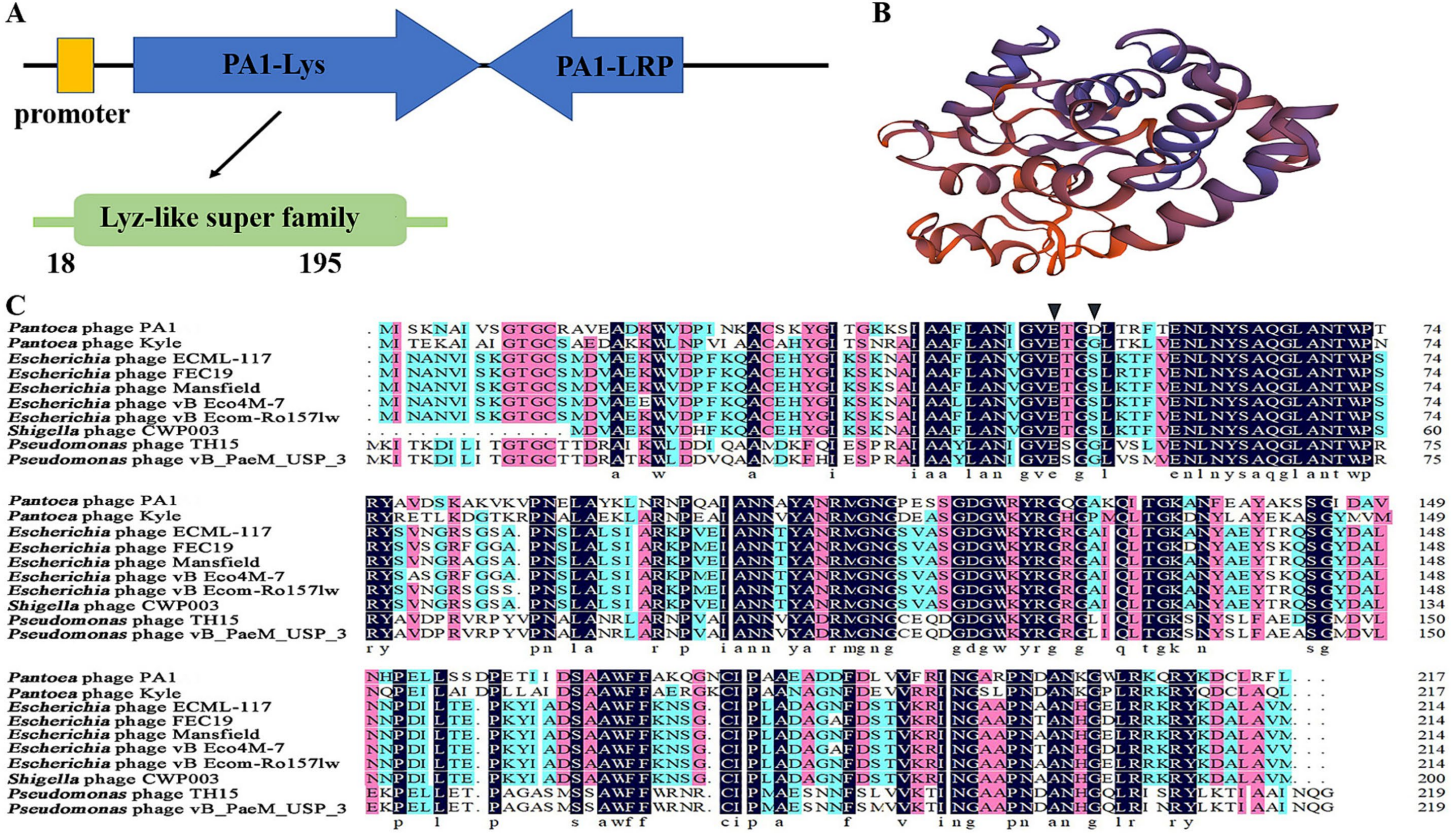
Bioinformatics and physicochemical properties of PA1-Lys**. (A)** Schematic diagram of the position of PA1-Lys in the phage PA1 genome. **(B)** 3D structure prediction of PA1-Lys. **(C)** Sequence alignment of PA1-Lys with that endolysin of *Pantoea* phage kyle (YP_009849889.1), *Escherichia* phage ECML-117 (YP_009101063.1), *Escherichia* phage FEC19 (YP_009813017.1), *Escherichia* phage Mansfield (QEG09877.1), *Escherichia* phage vB_Eco4M-7 (QFG06885.1), *Escherichia* phage vB_EcoM-Ro157lw (AVZ45696.1), *Shigella* phage CWP003 (UIS25194.1), *Pseudomonas* phage TH15 (QQO38587.1), and *Pseudomonas* phage vB_PaeM_USP_3 (QLI49345.1). The black arrow represents the predicted conserved motifs responsible for catalysis binding.

PA1-Lys contains 217 amino acids with a molecular weight of 23.7 kDa (Supplementary Table S1). PA1-Lys possess a lysozyme-like domain at amino acid site 18–195, belonging to the Lyz-like superfamily (Figure 4A). Bioinformatics analysis showed that the isoelectric point of PA1-Lys is 9.21 and the aliphatic index of PA1-Lys is 71.66. As a stable hydrophilic protein, PA1-Lys has 22 negatively charged residues and 29 positively charged residues. Moreover, PA1-Lys lacks a TMD domain structure or signal peptide. According to Phyre2 analysis, PA1-Lys consists of 1 beta-sheets and 8 alpha-helices. So we believed that PA1-Lys has a spiral structure (Figure 4B). Besides, Phyre2 analysis showed PA1-Lys has the most similar protein structure to *Salmonella Typhimurium* bacteriophage spnls endolysin. The 208 residues (96% of PA1-Lys) have been modeled with 99.5% confidence by the single highest scoring template. NCBI blastp results showed that nine proteins have the most similar genetic relationship with PA1-Lys. Both of them are endolysins from *Pantoea* phage kyle (YP_009849889.1), *Escherichia* phage ECML-117 (YP_009101063.1), *Escherichia* phage FEC19 (YP_009813017.1), *Escherichia* phage Mansfield (QEG09877.1), *Escherichia* phage vB_Eco4M-7 (QFG06885.1), *Escherichia* phage vB_EcoM-Ro157lw (AVZ45696.1), *Shigella* phage CWP003 (UIS25194.1), *Pseudomonas* phage TH15 (QQO38587.1) and *Pseudomonas* phage vB_PaeM_USP_3 (QLI49345.1) (Chen et al., 2021; D’Souza et al., 2019; Liao et al., 2019; Necel et al., 2020). Align the amino acid sequence of PA1-Lys with the amino acid sequences of the nine similar protein and results showed that the amino acid sequence of PA1-Lys is different from that of the 9 proteins.

Due to NCBI’s conservative domain prediction, no conserved motifs responsible for catalysis or substrate binding were identified. Generally speaking, the catalytic motifs of phage endolysins typically include amino acid residues in the active site, such as glycine (G), glutamic acid (E), serine (S), and aspartic acid (D), which directly participate in the enzyme’s catalytic reaction (Schmelcher et al., 2012; Vázquez et al., 2018). We speculated that the catalytic binding of PA1-Lys are E50 and D53, while the catalytic binding of other phages endolysin are E50 and G53 or E53 and S53. The differences in catalytic motifs among endolysins suggested that PA1-Lys may affect the structure and stability of bacterial cell walls through different mechanisms, which can lead to variations in their lytic efficacy and specificity against bacteria (Figure 4C).

### 3.6 Lytic activity of PA1-Lys

**Figure 5:**
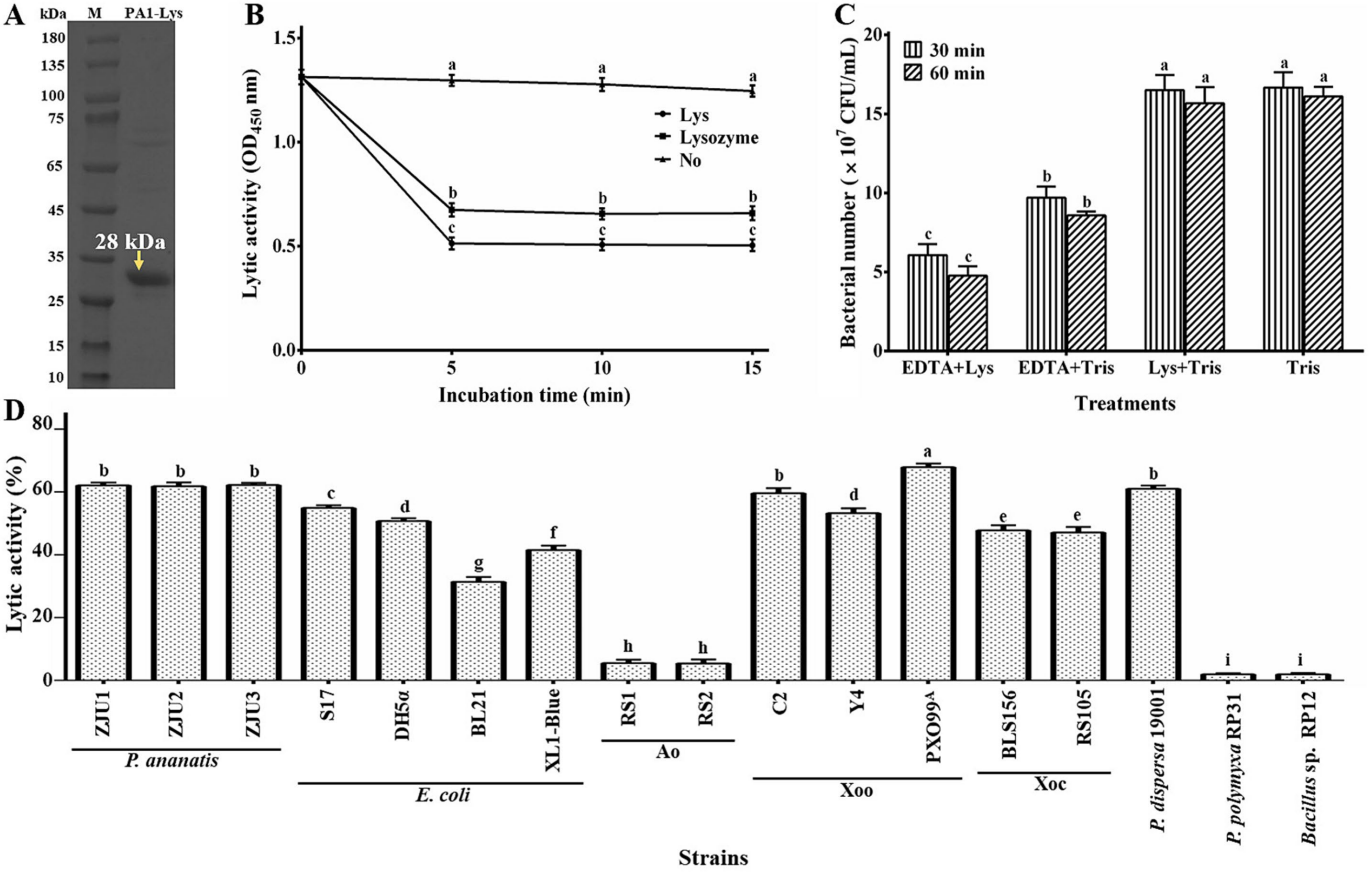
Lytic activity of PA1-Lys. **(A)** *In vitro* expression of PA1-Lys **(B)** Lytic effect of PA1-Lys on chloroform-pretreated *P. ananatis* strains ZJU1. **(C)** Lytic effect of PA1-Lys combined with EDTA. **(D)** Lytic spectrum of PA1-Lys. The error bars shows the mean of three replicates (*n* = 3 ± standard deviation). Different letters indicates significant differences in lytic activity among the different treatment groups at the same time point (*p* < 0.05).

Our research found that the PA1-Lys mainly exists in the lysate supernatant. As shown in Figure 5A, the size of PA1-Lys is about 28 kDa, which is consistent with the results obtained from the bioinformatics analysis. Previous studies have shown that the cell wall of Gram-negative bacteria has an outer membrane with a lipopolysaccharide layer, which prevents endolysin from entering the peptidoglycan layer, thus preventing endolysin from lysing bacteria (Kim et al., 2020). Outer membrane permeabilizers (chloroform and EDTA) can facilitate endolysin enter the bacteria (Shen et al., 2023). Previous study have reported that phage endolysin has a certain specificity (Skorynina et al., 2020). After adding chloroform, there was no significant change in the OD_450_ of the bacteria. However, after adding PA1-Lys for 5 min, the OD_450_ value of bacteria treated with chloroform decreased by 61.59%, and lysozyme (positive control) reduced the OD_450_ value of bacteria treated with chloroform by 52.40%. However, the OD_450_ value of bacteria pretreated with chloroform did not change after adding 0.1 M PBS (Figure 5B). Besides, EDTA also enhanced the inhibitory effect of PA1-Lys on the *P. ananatis* strain ZJU1. EDTA alone decreased bacterial count by 41.92%, while the combination of EDTA and PA1-Lys reduced bacterial count by 63.65% (Figure 5C). PA1-Lys can effectively destroy a variety of Gram-negative bacteria pretreated with chloroform, such as *P. ananatis*, *P. dispersa*, *E. coli*, Xoo, and Xoc, but it was unable to lyse Ao. Notably, PA1-Lys can not lyse Gram-positive bacteria (*Bacillus* and *P. polymyxa*) that do not require chloroform pretreatment (Figure 5D).

### 3.7 Stability of PA1-Lys

**Figure 6:**
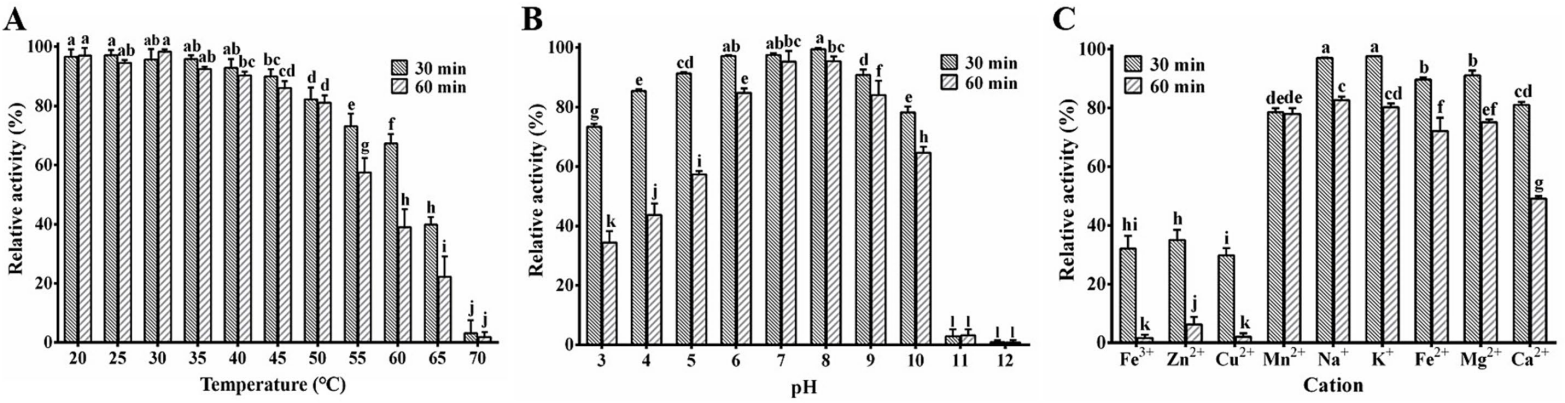
Stability of PA1-Lys. **(A)** Thermal stability of PA1-Lys. **(B)** pH stability of PA1-Lys. **(C)** Cation stability of PA1-Lys. The error bars stands for the mean of three replicates (*n* = 3 ± standard deviation). Different letters represents significant differences in the stability of PA1-Lys caused by different treatments at the same time point (*p* < 0.05).

The study on the thermal stability of PA1-Lys revealed robust lytic activity (>80%) across temperatures ranging from 20°C to 50°C (Figure 6A). However, the activity of PA1-Lys decreased notably with higher temperatures, showing a 96.83% decrease after incubation at 70°C for 30 min. Temperature exposure between 55°C and 65°C also significantly impacted PA1-Lys activity over time. Regarding pH stability (Figure 6B), PA1-Lys exhibited strong lytic activity across the pH range of 5 to 10. Optimal lytic activity was observed at pH 8, where PA1-Lys displayed 99.47% activity after 30 min and 95.4% after 60 min. The duration of treatment notably affected PA1-Lys activity, particularly at pH levels 3 to 6, where longer treatment times (60 min) resulted in decreased lytic activity compared to shorter treatments (30 min).

Furthermore, the study investigated the impact of cations on PA1-Lys activity (Figure 6C). Treatment with Fe^3+^, Zn^2+^, and Cu^2+^ for 30 min or 60 min significantly reduced PA1-Lys lytic activity by more than 75%. In contrast, Na^+^ and K^+^ did not affect PA1-Lys activity. However, prolonged exposure to these metal ions diminished PA1-Lys lytic activity. Interestingly, Ca^2+^ treatment initially showed no significant impact on PA1-Lys activity after 30 min but led to a significant decrease after 60 min. These findings underscore the sensitivity of PA1-Lys to temperature, pH, and specific cations, highlighting its potential application in environments where these factors can be controlled to optimize its efficacy as a biocontrol-agent.

### 3.8 Bacterial lysis function of PA1-Lys and PA1-LRP

Through bioinformatics analysis, we found that PA1-Lys does not possess a TMD or signal peptides, indicating that it might require the assistance of other proteins to facilitate its transmembrane transport, leading to cell lysis. Previous studies have reported that holin can assist endolysins in cell lysis (Bai et al., 2020; Chandran et al., 2022). However, we could not identify genes encoding holin through bioinformatics analysis. As we all know, lytic related proteins, such as holins, are located near the endolysins (Wang et al., 2022). PA1-Lys is expressed by the ORF12 gene of the phage PA1. Therefore, we chosed the proteins PA1-LPP and PA1-LRP expressed by ORF11 and ORF13 genes for the next research. In addition, studies have shown that some lytic related proteins contain TMD (Aslam et al., 2021). TMHMM analysis showed that among the 20 proteins near PA1-Lys, only the PA1-LVP contains three TMDs. Besides, the analysis of TMHMM showed PA1-LRP contains a hydrophobic region within the 40–60 amino acid range, while the PA1-LPP has no hydrophobic region. Then PA1-LRP, PA1-LPP, and PA1-LVP were fused and co-expressed with PA1-Lys to investigate whether the three fusion proteins could lyse bacteria. After 24 h of IPTG induction, we observed the OD_600_ value of 28a-Lys-LRP was 68.88% lower compared to 28a-Lys, declaring that the PA1-LRP can help PA1-Lys perform cell lysis functions. Therefore, ORF13 expressed protein PA1-LRP drew my attention.

According to Phyre2 analysis, we found that PA1-LRP has 2 alpha-helices and no beta-sheets. In addition, 3D structure prediction of PA1-LRP was showed in Supplementary Figure S3. Through comparison, PA1-LRP does not resemble any known protein structures in the Protein Data Bank (PDB). Through NCBI blastp, the similar proteins of PA1-LRP are all hypothetical protein. *Pantoea* phage Kyle hypothetical protein HWC52 gp058 has the highest similarity with PA1-LRP, with a query coverage of 97%. Besides, bioinformatics analysis results indicated that the size of the PA1-LRP is 8.5 kDa.

**Figure 7:**
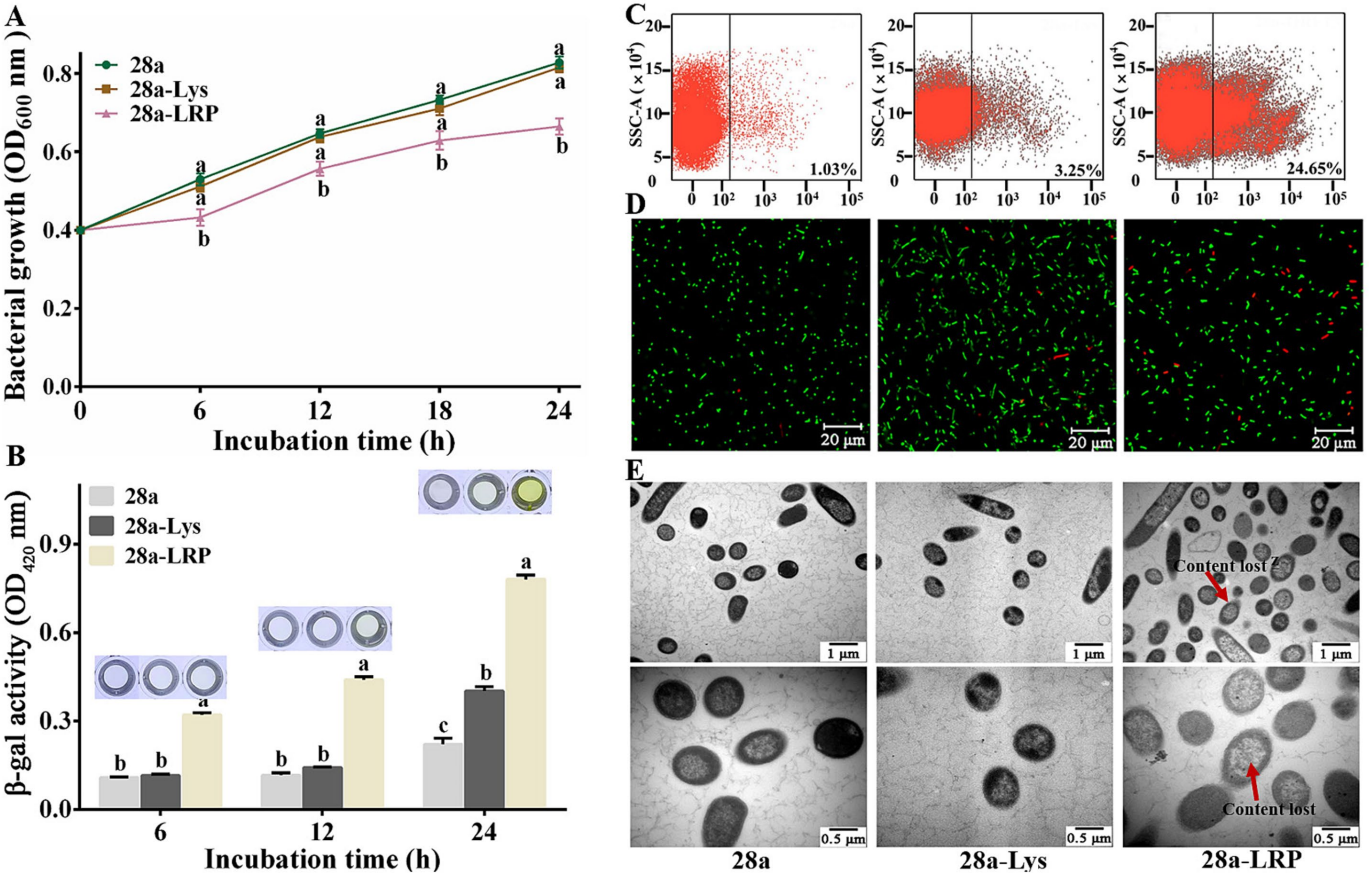
Effects of PA1-Lys and PA1-LRP on cell. **(A)** Bacterial growth curve. The statistically significant results are based on the OD_600_ values of 28a, 28a-Lys and 28a-LRP induced at the same induction time. **(B)** Determination of *β*-galactosidase in bacterial supernatant. The statistically significant results are based on the OD_420_ values of 28a, 28a-Lys and 28a-LRP induced at the same induction time. **(C)** Flow cytometry analysis. **(D)** Live/dead cell staining analysis. **(E)** TEM images of bacterial ultrastructure. The red arrow represents the lost of cell contents. Magnification was 20,000 x and 40,000 x, respectively. The error bars stands for the mean of three replicates (n = 3 ± standard deviation). Different letters represents significant differences caused by different treatments at the same time point (*p* < 0.05).

Bacterial growth assay makes it clear that after application of IPTG for 6, 12, 18, and 24 h, there was no significant difference in the OD_600_ values of 28a-Lys compared to 28a, while the OD_600_ values of 28a-LRP reduced by 13.40, 13.91, 14.18, and 19.67% (Figure 7A). The *β*-galactosidase assay measured the effect of PA1-Lys and PA1-LRP on cell membrane permeability. When the cell membrane is damaged, the β-galactosidase inside the cell membrane flow out, which can break down ONPG and produce yellow O-nitrophenol. Therefore, the more yellow the color of the bacterial supernatant, the greater the cell membrane permeability. After induction of IPTG for 24 h, the color of 28a was transparent, suggesting that the bacterial cell membrane was intact. However, the supernatants of 28a-Lys and 28a-LRP turned yellow, and the color of 28a-LRP was stronger than that of 28a-Lys, indicating that the cell membranes of 28a-Lys and 28a-LRP were unstable, with 28a-LRP having higher cell permeability (Figure 7B).

Further analysis of the mortality rates after 24 h of induction using flow cytometry revealed mortality rates of 3.25% for 28a-Lys and 24.65% for 28a-LRP, while the mortality rates of 28a was only 1.03% (Figure 7C). Utilizing a live/dead bacterial staining kit, live bacteria were stained green while dead bacteria appeared red. Fluorescence microscopy observations showed that all bacteria were short rod-shaped, with a higher proportion of dead bacteria observed in 28a-LRP compared to 28a (Figure 7D). To investigate the effects of PA1-Lys and PA1-LRP on the morphology of *E. coli* in greater detail, ultrastructural analysis using TEM was performed. TEM observations indicated that both 28a and 28a-Lys exhibited intact cell structures with high-density intracellular substances (Figure 7E). In contrast, 28a-LRP showed a relatively lower density of intracellular materials compared to 28a-Lys and 28a. These findings strongly suggest that PA1-LRP contributes to cellular damage, resulting in altered cell morphology and increased mortality rates among bacterial populations.

### 3.9 Effects of co-expression of PA1-Lys and PA1-LRP on cells

**Figure 8:**
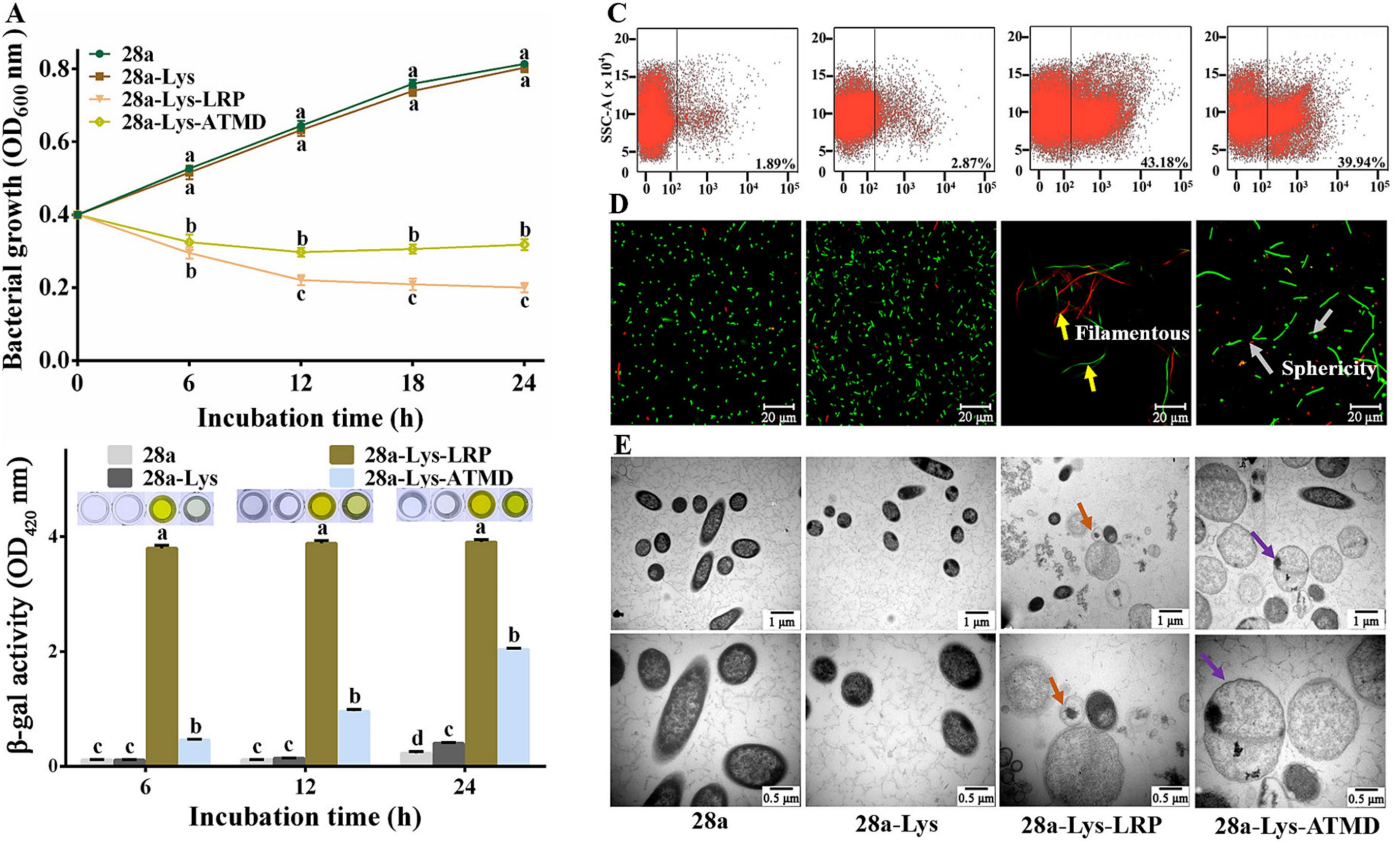
Effects of co-expression of PA1-Lys and PA1-LRP on cell. **(A)** Bacterial growth curve. The statistically significant results are based on the OD_600_ values of 28a, 28a-Lys, 28a-Lys-LRP and 28a-Lys-ATMD induced at the same induction time. **(B)** Determination of β-galactosidase in bacterial supernatant. The statistically significant results are based on the OD_420_ values of 28a, 28a-Lys, 28a-Lys-LRP and 28a-Lys-ATMD induced at the same induction time. **(C)** Flow cytometry analysis. **(D)** Live/dead cell staining analysis. The yellow arrows point to the bacteria becoming filamentous, while the gray arrows points to the bacteria becoming spherical. **(E)** TEM images of bacterial ultrastructure. The orange arrow represents the transformation of bacteria into vacuoles, and the purple arrow represents bacterial enlargement. The magnification was 20,000x and 40,000x, respectively. The error bars stands for the mean of three replicates (*n* = 3 ± standard deviation). Different Minuscule mean significant differences caused by different treatments at the same time point (*p* < 0.05).

Previous studies have shown that adding an exogenous TMD to endolysins can help them lyse cells (Wu et al., 2021). Therefore, we added an exogenous TMD at the C-terminus of PA1-Lys as a positive control (28a-Lys-ATMD). After 6, 12, 18, and 24 h of IPTG induction, the OD_600_ value of 28a-Lys-ATMD decreased by 38.21, 53.88, 59.68, and 60.89%, compared to the negative control of 28a while the OD_600_ value of 28a-Lys-LRP decreased by 43.92, 65.84, 72.46 and 75.40% (Figure 8A). After 6 h of induction, the supernatant of 28a-Lys-LRP turned dark yellow and showed a stronger color compare to 28a-Lys-ATMD (Figure 8B). Flow cytometry measurement results suggested that the mortality rates of 28a-Lys-LRP and 28a-Lys-ATMD were 43.18 and 39.94%, respectively (Figure 8C). Compared with the positive control, the co-expression of PA1-Lys and PA1-LRP possessed strong antibacterial properties.

To further elucidate the synergistic mechanism of PA1-Lys and PA1-LRP in bacterial lysis, we observed the morphological and structural changes of cells using fluorescence and TEM. The morphology of all bacteria in the 28a-Lys-LRP sample was filamentous, while the morphology of 28a and 28a-Lys were short rod-shaped. Moreover, some bacteria in the 28a-Lys-ATMD sample became spherical (Figure 8D). The field of view of the 28a-Lys-LRP samples contained relatively fewer bacteria and a higher proportion of dead bacteria, consistent with the bacterial growth experiments and flow cytometry results.

Compared with 28a and 28a-Lys, the cells of 28a-Lys-LRP and 28a-Lys-ATMD were enlarged, with lower intracellular material density, lighter cell color, and even some cells formed vacuoles due to complete loss of contents (Figure 8E). These results indicated that co-expression of PA1-Lys and PA1-LRP can effectively inhibit bacterial growth, ultimately leading to bacterial death. Moreover, PA1-LRP has a stronger auxiliary effect than exogenous TMD in helping PA1-Lys perform cell lysis functions. Due to the synergistic destructive effect of PA1-LRP and PA1-Lys on *E. coli*, we conducted the bacterial two-hybrid experiment to explore whether they directly interact *in vivo*. Positive colonies grew well on the selected culture medium. However, the colonies of *E. coli* XL1-Blue co-expressing PA1-LRP and PA1-Lys was unable to grow on the selected medium, consistent with the negative colonies. The bacterial two-hybrid experiment showed that there was no direct interaction between PA1-Lys and PA1-LRP (Supplementary Figure S2).

### 3.10 Effects of co-expression of PA1-Lys and HPP on cells

**Figure 9:**
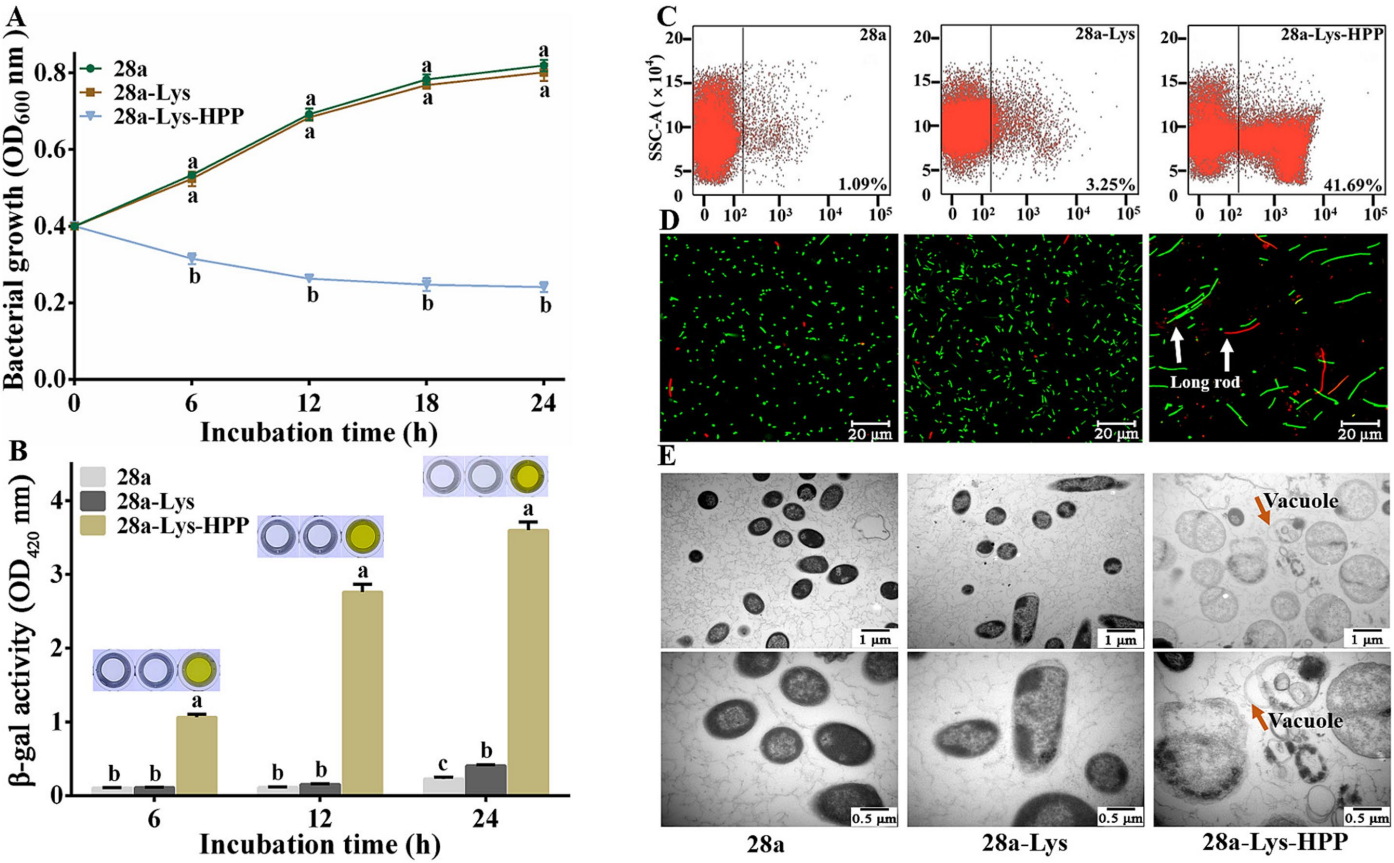
Effects of Co-Expression of PA1-Lys and HPP on cell. **(A)** Bacterial growth curve. The statistically significant results are based on the OD_600_ values of 28a, 28a-Lys and 28a-Lys-HPP induced at the same induction time. **(B)** Determination of β-galactosidase in bacterial supernatant. The statistically significant results are based on the OD_420_ values of 28a, 28a-Lys and 28a-Lys-HPP induced at the same induction time. **(C)** Flow cytometry analysis. **(D)** Live/dead cell staining analysis. The white arrow points to long rod-shaped bacteria **(E)** TEM images of bacterial ultrastructure. The orange arrow represents the transformation of bacteria into vacuoles. The magnification was 20,000x and 40,000x, respectively. The error bars stand for the mean of three replicates (*n* = 3 ± standard deviation). Different letters represent significant differences caused by different treatments at the same time point (*p* < 0.05).

Previous research has shown that fusion expression of endolysin and hydrophobic peptides can damage cells (Sitthisak et al., 2023). We engineered a fusion of HPP at the C-terminus of PA1-Lys to investigate its impact on PA1-Lys function. Following induction periods of 6, 12, 18, and 24 h, the OD_600_ values of 28a-Lys-HPP decreased by 39.77, 61.55, 67.84, and 70.16%, respectively, compared to 28a-Lys (Figure 9A). Notably, after just 6 h of induction, the supernatant of 28a-Lys-HPP exhibited a yellow color (Figure 9B), suggesting cellular disruption and metabolic changes. Flow cytometry analysis revealed a 38.44% higher mortality rate in 28a-Lys-HPP compared to 28a-Lys (Figure 9C). Fluorescence microscopy observations further supported these findings, revealing predominantly long rod-shaped bacteria in 28a-Lys-HPP samples, accompanied by a lower total bacterial count and increased presence of dead bacteria relative to 28a-Lys (Figure 9D). TEM imaging of 28a-Lys-HPP samples depicted significant cellular content leakage, vacuole formation, and substantial cell debris, similar to observations in 28a-Lys-LRP samples (Figure 9E). These results collectively demonstrate that HPP enhances PA1-Lys’s ability to disrupt cell membranes, leading to effective bacterial cell destruction.

## 4 Discussion

In recent years, *P. ananatis* has been recognized as a new type of pathogenic bacteria affecting various plants, and is widely distributed in nature (Xiao et al., 2022; Xu et al., 2021). As natural enemies of bacteria, phages can not only specifically recognize and effectively kill target bacteria, but also co-evolve with the host, demonstrating excellent biosafety (Oyejobi et al., 2023). Therefore, phages are very important for the treatment of pathogenic bacteria. However, despite their potential use, research on *P. ananatis* phage is limited.

In this study, we isolated a phage with specific lysis capabilities against *P. ananatis* from rice leaves and named it PA1. The whole genome sequencing of phage PA1 showed relatively low similarity to other phages in the NCBI database. Although different annotation tools were used to annotate the PA1 phage proteins, there are still many hypothetical proteins among the 105 putative open reading frames, which may be related to the limited number of reported *Pantoea* phages.

However, the evolutionary tree indicated a close genetic relationship between phage PA1 and *Pantoea* phages. TEM observation confirmed that phage PA1 has an icosahedron head and a contractile tail, classifying it under the *Chaseviridae* family. Previous studies have shown that phages with larger burst sizes tend to have more effective lysis effects (Manohar et al., 2018). The results of the one-step growth experiment showed that phage PA1 has a latent period of 40 min and a burst size of 17.17, indicating its potential as a biocontrol agent against diseases caused by *P. ananatis*. As a biocontrol agent, phages should remain stable under various environmental conditions, including pH and temperature (Liu et al., 2021). Phage stability testing showed that phage PA1 was stable within a pH range of 6 to 8 and a temperature range of 4 to 60°C, signifying its suitability for biological control.

However, phage therapy faces several obstacles, including the limitations in phage infection spectrum and the development of bacterial resistance to phages (Hatfull et al., 2022). Phage endolysins are peptidoglycan hydrolases encoded by phages and synthesized by host bacteria (Li et al., 2021; Zhang et al., 2022). Due to their broad lytic spectrum, phage endolysins have emerged as potential novel antibacterial agents (Wan et al., 2021). In our study, we analyzed the sequencing data of phage PA1 and identified the phage endolysin PA1-Lys. PA1-Lys can break down various chloroform-treated bacteria, including *P. ananatis*, *P. dispera,* Xoo, Xoc and *E. coli*. Also, PA1-Lys remaines stable under different pH levels (6 to 10) and temperatures (20 to 50°C).

In this study, the PA1-Lys cannot directly lyse cells. Bioinformatics analysis showed that PA1-Lys possesses a Lyz-like superfamily domain. Notably, unlike Gram-positive bacteria, Gram-negative bacteria have an outer membrane containing lipopolysaccharide, which makes it impossible for the endolysins lacking transmembrane and SAR domains to directly hydrolyze the bacterial cell wall through the outer membrane. Therefore, the endolysins of Gram-negative bacteria cannot directly penetrate the bacteria (Rahman et al., 2021). Studies have shown that the addition of EDTA and chloroform can disrupt the outer membrane of Gram-negative bacteria, thereby helping endolysins to destroy bacteria (Legotsky et al., 2014; Nie et al., 2022; Wang et al., 2024). Consequently, this study confirmed that PA1-Lys exhibits enhanced lytic activity in the presence of EDTA and chloroform.

Phage holins can form pores in the bacterial cytoplasmic membrane at specific times, (Xu et al., 2021) facilitating the endolysin to damage cell wall peptidoglycan and leading to rapid dissolution of the host cells (Zhou et al., 2021). Previous studies have shown that the transport function of holins may be determined by their TMD (Wu et al., 2021). In this study, an exogenous TMD with PA1-Lys was fused and the fusion protein indeed caused cell lysis. Since PA1-Lys alone was unable to directly lyse bacteria, we speculated whether the presence of a holin to assist in its lysis effect. However, no holin gene was observed through RAST analysis. Previous studies have shown that some lytic related proteins, such as holins, located near the endolysins and contain at least one TMD (Guo et al., 2022; Lu et al., 2020). TMHMM analysis showed that among the 20 proteins near PA1-Lys, only the PA1-LVP contains three TMDs. PA1-Lys is expressed by the ORF12 gene of the phage PA1. Therefore, we also chosed the proteins PA1-LPP and PA1-LRP expressed by ORF11 and ORF13 genes for the next research. We fused PA1-Lys with PA1-LPP, PA1-LRP, and PA1-LVP for expression, and the results showed that only PA1-LRP fused with PA1-Lys can inhibit bacterial growth. Additionally, we found PA1-LRP was found to inhibit bacterial growth and cause cell death. Moreover, the lytic effect of co-expressing PA1-LRP with PA1-Lys was significantly stronger than expressing PA1-LRP or PA1-Lys alone. After 24 h, the OD_600_ value of 28a-Lys-LRP was 0.444 lower than that of 28a-LRP. In addition to inhibiting bacterial growth, the co-expression of PA1-LRP with PA1-Lys altered the bacterial shape from short rod to filamentous, suggesting a potential inhibitory effect on bacterial cell division. Thus we consider PA1-LRP to be a novel lysis-related protein. Bioinformatics analysis indicated that PA1-LRP contains a hydrophobic region. Previous studies have shown that hydrophobic regions assist endolysins in degrading bacterial cells (Ning H. Q. et al., 2021; Xu et al., 2021). *E. coli* phages endolysin Lysep3 modified with hydrophobic amino acids can undergo external lysis of *E. coli* (Yan et al., 2019). Hydrophobic fusion of intracellular endolysin (lysAB-vT2 fusion) can dissolve the cell wall of Gram-negative bacteria (Sitthisak et al., 2023). The co-expression of the HPP with PA1-Lys also led to cell death. Therefore, PA1-LRP, using HPP, can assist PA1-Lys in disrupting bacterial cells.

## 5 Conclusion

In summary, this study successfully isolated and characterized *Pantoea* phage PA1 belonging to the *Chaseviridae* family. Phage PA1 exhibited promising characteristics as a biocontrol agent, including a large burst size of 17.17 phages per infected cell and stability over a range of pH and temperature conditions. Genomic analysis identified the endolysin PA1-Lys, which showed broad lytic activity against various chloroform-treated Gram-negative bacteria. Importantly, this research uncovered a novel lysis-related protein, PA1-LRP, which enhanced the bactericidal activity of PA1-Lys. Co-expression of PA1-Lys with PA1-LRP resulted in significant bacterial growth inhibition, cell shape alterations, and increased cell death compared to either protein alone. The HPP was found to be crucial in assisting PA1-Lys to disrupt bacterial cells. These findings contribute to our understanding of phage lysis mechanisms and offer new strategies for combating bacterial infections. The synergistic effect of PA1-Lys and PA1-LRP presents a promising approach for developing more effective antimicrobial agents. Further research into the precise mechanisms of PA1-LRP lytic activity and its potential applications could lead to novel therapeutic interventions against antibiotic-resistant pathogens.

## Data Availability

The original contributions presented in the study are included in the article/Supplementary material, further inquiries can be directed to the corresponding authors.

## Footnotes

^1^ http://www.bcgsc.ca/platform/bioinfo/software/abyss

^2^ http://rast.nmpdr.org/rast.cgi

^3^ http://topaz.gatech.edu/GeneMark/genemarks.cgi

^4^ https://github.com/gbouras13/Pharokka

^5^ http://blast.ncbi.nlm.nih.gov/

^6^ http://lowelab.ucsc.edu/tRNAscanSE/

^7^ https://proksee.ca/projects/

^8^https://web.expasy.org/protparam/; accessed on: 30 December 2021

^9^https://www.ncbi.nlm.nih.gov/Structure/cdd/wrpsb.cgi; accessed on: 30 December 2021.

^10^https://services.healthtech.dtu.dk/services/TMHMM-2.0; accessed on: 30 December 2021.

^11^https://services.healthtech.dtu.dk/services/SignalP-5.0; accessed on: 30 December 2021.

^12^https://swissmodel.expasy.org; accessed on: 30 December 2021.

^13^ http://www.sbg.bio.ic.ac.uk/phyre2/phyre2_output/41b500971e497a23/summary.html
