# Supplementary material for: Role of hypothetical protein PA1-LRP in antibacterial activity of endolysin from a new Pantoea phage PA1
Source: Front Microbiol. 2024 Oct 23;15:1463192. doi: 10.3389/fmicb.2024.1463192 (PMC11538084; doi:10.3389/fmicb.2024.1463192)
Supplement: Supplementary file 1 [file Data_Sheet_1.docx]

**Table S1. Analysis of amino acid of PA1-Lys and PA1-LRP**

|  | PA1-Lys | | PA1-LRP | |
| --- | --- | --- | --- | --- |
| Amino acid | Number | Proportion (%) | Number | Proportion (%) |
| Ala (A) | 29 | 13.4 | 5 | 6.80 |
| Arg (R) | 12 | 5.50 | 10 | 13.70 |
| Asn (N) | 19 | 8.80 | 0 | 0.00 |
| Asp (D) | 13 | 6.00 | 3 | 4.10 |
| Cys (C) | 4 | 1.80 | 1 | 1.40 |
| Gln (Q) | 6 | 2.80 | 3 | 4.10 |
| Glu (E) | 9 | 4.10 | 7 | 9.60 |
| Gly (G) | 18 | 8.30 | 3 | 4.10 |
| His (H) | 1 | 0.50 | 0 | 0.00 |
| Ile (I) | 13 | 6.00 | 5 | 6.80 |
| Leu (L) | 12 | 5.50 | 12 | 16.40 |
| Lys (K) | 17 | 7.80 | 4 | 5.50 |
| Met (M) | 2 | 0.90 | 1 | 1.40 |
| Phe (F) | 8 | 3.70 | 1 | 1.40 |
| Pro (P) | 9 | 4.10 | 2 | 2.70 |
| Ser (S) | 13 | 6.00 | 4 | 5.50 |
| Thr (T) | 9 | 4.10 | 4 | 5.50 |
| Trp (W) | 5 | 2.30 | 0 | 0.00 |
| Tyr (Y) | 8 | 3.70 | 3 | 4.10 |
| Val (V) | 10 | 4.60 | 5 | 6.80 |

**Table S2.** Gene sequence of PA1-Lys, PA1-LRP, PA1-LPP, PA1-LVP,ATMD, HPP, Lys-ATMD, and Lys-HPP

| Gene | Nucleotide Sequence (5'–3') |
| --- | --- |
| PA1-Lys | ATGATCAGTAAAAACGCAATTGTATCAGGAACAGGATGCAGAGCAGTAGAAGCTGATAAATGGGTTGATCCTATCAATAAAGCTTGCAGCAAATACGGCATCACTGGTAAAAAGTCAATCGCCGCATTCCTCGCTAACATTGGAGTTGAGACGGGTGATCTTACTCGTTTTACCGAAAACCTGAATTATAGCGCACAGGGGCTGGCTAACACATGGCCTACGCGTTACGCGGTTGATAGTAAGGCGAAAGTGAAGGTTCCTAACGAGCTGGCGTATAAGCTTAATCGCAACCCACAGGCCATTGCCAACAACGCCTATGCAAACCGCATGGGCAACGGCCCTGAGTCGAGCGGAGACGGCTGGCGCTATCGCGGACAGGGCGCCAAACAGATCACCGGAAAGGCCAACTTCGAAGCCTATGCAAAATCGTCGGGCATTGATGCCGTAAACCATCCAGAGCTGCTTAGCTCCGACCCTGAAACGATCATCGATAGCGCGGCTTGGTTCTTCGCTAAGCAGGGGAATTGCATCCCCGCCGCAGAAGCTGACGACTTTGATCTGGTAGTTTTTCGCATCAACGGAGCGCGACCTAACGACGCCAACAAGGGCTGGTTGCGTAAGCAGCGTTATAAAGATTGCTTGAGATTCTTATAA |
| PA1-LRP | ATGGTAAGGCGCAGACGAAGAACAGATGTTGAAGAGACAAGGCTGAGGAAAGAGTTATTAAGCTCTATTCTCAGCCTTCTTGTTAGACTTATTGGCGAGACGGTAAAGGACAGGTTGGCAAGGACTATTTTGATCAGCTGTATTGGCGCTTTCGGTTATTACTATGACTTAGCGCCTGCTGAACTTGAACCTGCTCTCCAGAAAGTAGAACAGCAAAAGTAA |
| PA1-LPP | ATGGTTCAAATCTACGTCAGGCCGTTTGCAGAGAGTGGTGATAAAACCAATCCACCAGACAACGACAACGCCGGATTTGTCAGCTTTACTCAAGGTTATACAGCAGACTATGAAATAAATCTCGCTGCAAATAACCCTCAAGCTAAAGCAGTTGAACGTCAAGTTCAAAACGCATTGTTCAATGGCATAACTGATAACTTACTTTGGTATCAGAGCCACGGCTTTATGCAATATAGCAATACAATCACTTATCAATTAAATGATATGGTGGTAGATAATACTGGCTCAACAGGCTCTCCAGTTTGGGAAATATTCAGAAGTCGAATAAACAACAACGGAAACAAGCCTAGTACAGTTAGCCAGAATACGCCTTCACCAACCTGGGAAAAGATACCATTACCAAGCGCAATCAAGAGTATGATTCCTTTCCCTGCTGGCGGCGACCTTATGAACGTAGGAACGCCAGTGGCATCACTCCTTCTTTCTGGCGCAAACCTTAATAATTTGGCTCGTGGATTCTTTGTCGCGCCAAATCCTGCTTCTGTATCGGGAATCGTTAACAGGCCTAATGCTGTCATTGCTTCGAATGCCCCTTTCACACTTGAACAGTGCGATTGGACAACTAACGACGGCTCTGTAGCAAACTACAAACTACAGCGCGTTGTTACTATTGACGGAAACATCTTCATCCGTTCTTCACGTAATAACGCCTGGTCTAACTGGACACAACAGGCCACAGCTAACGATACGCAAGGCGGAATATTTAACTTCGCCCAGGGTTCAAGTTCTACGCCAGCTCAGAGTGTATCATTGACGCCTTTGCTGGGTATCGCAACCCTTCGCGATGGCATGATTATTCGCTTCGTCGCTTTAGCAGATAACACTGGGCCATTCAGCGTTACAATTGCTAACGCAACAACAGAGGTCTTAGGGCGCGACCTTTTATCTCTTCCTGCTGGTGCTATCATTGCAAACCAGATTGTGGAAATAATCTACCGAAACGGTCGCTGGCACCTTCTTTCTTCAATGGGCGGCCAGTTCGTTGTTCCATTAGCGATAGCCGACAATGCCGCGCCAACTTGGAAACAGGTCAAAGACTTATTCGCCACTGCGCTTAACTTTGATAAAATTTTCCCTGTTGGATTTATCATCGGAATGGCGAACGGAACCAACCCTAACGCTGCTTTCCCACCCACTGTCTGGACTAGAATTCCAGATGGCGTTGTTTTGAGAACAGTAGCCGCAGGCCAATCAGCTGCCAATGTCTATGGAAACGATAACGTAACCCTAACGATGCAGAATATGCCTTTGCACTCGCATGGGGTTAACATCGCTACCAACGAGCAGCCTTCATATCAAGTGCAAACTCAAGACACAGATTTGGGGCAGAGGTCGACTAGCGAAAGCGGATGGCACGGCCATGGCTTTAGCGGAACAACCAACCCAGGCGGATACCACGGACACTCAGCTTACGCCGATACGCAAGGCGCTCACGCTCACGCCTTTGTAGGCGATGACCAGTTGACGGCATATTATGGCGTGGCACAATATCGCGTTTCAAACTATGACGCTCGCTCTGATTCTGGCGGAATGGCAAACCTTTATTGGACGTCGACCGCAGGACAACATAGTCACAACATCGGCGTAAACGCTGGCGGAGAGCACCAGCACACCTACGGCGGAAATACCGAAGGCAGCGGCAATCACACTCACACATTGACAATAGGTGCTCACCGACACACACTGACAATTCCGGCGCACAGCCACCAAGTTGTTGGCGATACCGCGAGCGCAGGTCAATCAAATCCAAGTTCTATTTCAGTCGTTCAGCGCCACTTTGCGATGGTCGGCTGGTTCCGTTCTAGTTAA |
| PA1-LVP | ATGAACTGGCAAGACATTGGCAAGGCAGTAGGTGGCGCGGCTCCAATCGTAGGTGGCCTTTTAGGTGGCCCAGCAGGTGCAGCCATAGGTAATATCGTTGCGTCGACGTTAGGCGTTAATAACACGCCTGACAGTGTAAGCGACGCACTTAAAAACAATCCAGACGCTCTCGTTAAAATCCAAGAGCTGCAAACCAACTCCAAAGTGGAACTTCAGAGGCTGGCTGTATTGGCCGAACAACACAGGATGGAAGCTGAGCTTCTTCAGTATCAAGCGGAAGCAGGCGACAGGGCTAACGCTCGTGAACTTGCCGCGAAACAGCCTAATGATCGAATTCGTCCTACAATCACAATTATGATGATTTTGGGCGCAATAGCTATTGTCTTTTTAGTTTTCAGCGGCTATAGTAAAGAACTGCTGACCAATGCAACGGCTTCGCTCACTGTGGGCACTGTTATGGGGTATTGGTTCGGCGAAGTGAAACAGGTAATGGCTTTCTGGTTCGGAACGACAAAGGACGCCGGAACACAGAGCAACACGATTACCCGTTTCGCTGTTTCACCTGACAAAATCGTCGAAAAACCTGGAAATGAGGATAAAAAGTAAATGAGCAATATTTTCGTAATTTTCGCCCATATCCTTGCGTGGTCAAACGCTCGGAACATTATCAATGGCACTACCGCCGTTGATCAGTTCGTCAAGCTGGTTAGCGAGTTCTCGAACGAAATCGCCGAAGCTCTTTATGAAATTGCCGCCTATTCTGGCGGTGACGTTGAAGAGCGTAACGCGATTATCCATACGAAGTTGGCCGATGCAATCGGCGATTCGTTGGTAGTTCTAGTTAATGTTTGTGCACAACGCGGTATTGACATTGAAAGCGTTTGGCAATCCCAACACTTCCCCGTCTACAATCAGGTGCCAAATTACGGCTTTCTCGTTGCTTCGATGGGCCAGGGCAGGCTGGCTGATGCTCTTAAGAAGAACAACCAAGACGATATCTATTCTGCCATCGGCACAATGTTATACGGGCTTGAGTTGATCTGCGAAGAATACACTTTAAGTCCTCGCGGTTGCGCTCAAGATGCGTACAACGAAATCAAAGACCGTAAAGGCGTCGTTTATAACGGCATCTTTGTAAAGTCTGATGACGCCAACTATGAACGTATTTTGGCTGAGTTAGGTCTACCGTTATAA |
| ATMD | AGCCTCGGCAACTGGCTGCTCCCCGTGCTGCTGGTGGCGGTGGCCGCCCTGTGCGCCTACATCGTCTGGGAGCGTGTGAAGGTGCGGCGAGAGGGGTGGTCA |
| HPP | AGGACTATTTTGATCAGCTGTATTGGCGCTTTCGGTTATTACTAT  GACTTAGCGCCTGCT |
| Lys-ATMD | ATGATCAGTAAAAACGCAATTGTATCAGGAACAGGATGCAGAGCAGTAGAAGCTGATAAATGGGTTGATCCTATCAATAAAGCTTGCAGCAAATACGGCATCACTGGTAAAAAGTCAATCGCCGCATTCCTCGCTAACATTGGAGTTGAGACGGGTGATCTTACTCGTTTTACCGAAAACCTGAATTATAGCGCACAGGGGCTGGCTAACACATGGCCTACGCGTTACGCGGTTGATAGTAAGGCGAAAGTGAAGGTTCCTAACGAGCTGGCGTATAAGCTTAATCGCAACCCACAGGCCATTGCCAACAACGCCTATGCAAACCGCATGGGCAACGGCCCTGAGTCGAGCGGAGACGGCTGGCGCTATCGCGGACAGGGCGCCAAACAGATCACCGGAAAGGCCAACTTCGAAGCCTATGCAAAATCGTCGGGCATTGATGCCGTAAACCATCCAGAGCTGCTTAGCTCCGACCCTGAAACGATCATCGATAGCGCGGCTTGGTTCTTCGCTAAGCAGGGGAATTGCATCCCCGCCGCAGAAGCTGACGACTTTGATCTGGTAGTTTTTCGCATCAACGGAGCGCGACCTAACGACGCCAACAAGGGCTGGTTGCGTAAGCAGCGTTATAAAGATTGCTTGAGATTCTTAAGCCTCGGCAACTGGCTGCTCCCCGTGCTGCTGGTGGCGGTGGCCGCCCTGTGCGCCTACATCGTCTGGGAGCGTGTGAAGGTGCGGCGAGAGGGGTGGTCA |
| Lys-HPP | ATGATCAGTAAAAACGCAATTGTATCAGGAACAGGATGCAGAGCAGTAGAAGCTGATAAATGGGTTGATCCTATCAATAAAGCTTGCAGCAAATACGGCATCACTGGTAAAAAGTCAATCGCCGCATTCCTCGCTAACATTGGAGTTGAGACGGGTGATCTTACTCGTTTTACCGAAAACCTGAATTATAGCGCACAGGGGCTGGCTAACACATGGCCTACGCGTTACGCGGTTGATAGTAAGGCGAAAGTGAAGGTTCCTAACGAGCTGGCGTATAAGCTTAATCGCAACCCACAGGCCATTGCCAACAACGCCTATGCAAACCGCATGGGCAACGGCCCTGAGTCGAGCGGAGACGGCTGGCGCTATCGCGGACAGGGCGCCAAACAGATCACCGGAAAGGCCAACTTCGAAGCCTATGCAAAATCGTCGGGCATTGATGCCGTAAACCATCCAGAGCTGCTTAGCTCCGACCCTGAAACGATCATCGATAGCGCGGCTTGGTTCTTCGCTAAGCAGGGGAATTGCATCCCCGCCGCAGAAGCTGACGACTTTGATCTGGTAGTTTTTCGCATCAACGGAGCGCGACCTAACGACGCCAACAAGGGCTGGTTGCGTAAGCAGCGTTATAAAGATTGCTTGAGATTCTTAAGGACTATTTTGATCAGCTGTATTGGCGCTTTCGGTTATTACTATGACTTAGCGCCTGCT |





**Fig. S1.** The effect of co-expressing PA1-Lys and its nearby proteins on bacterial growth. The error bars stands for the mean of three replicates (n = 3 ± standard deviation). Different Minuscule mean significant differences caused by different treatments at the same time point (*p*<0.05).


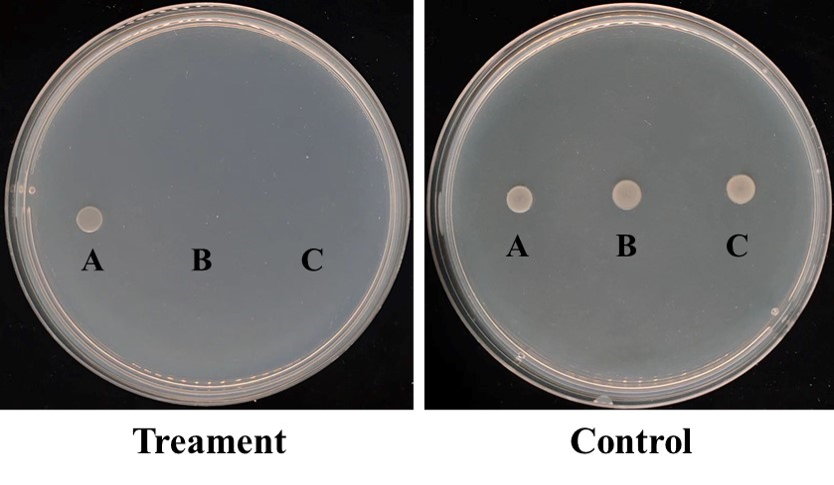


**Fig. S2.** Bacterial two-hybrid assay for analysis of PA1-Lys and PA1-LRP interaction. A: The *E.coli* XL1-Blue contains PBT-LGF2 and PTRG-Gal11^P^; B: The *E.coli* XL1-Blue contains the PBT-Lys vector and PTRG-LRP vector; C: the *E.coli* XL1-Blue contains PBT and PTRG is the negative control.


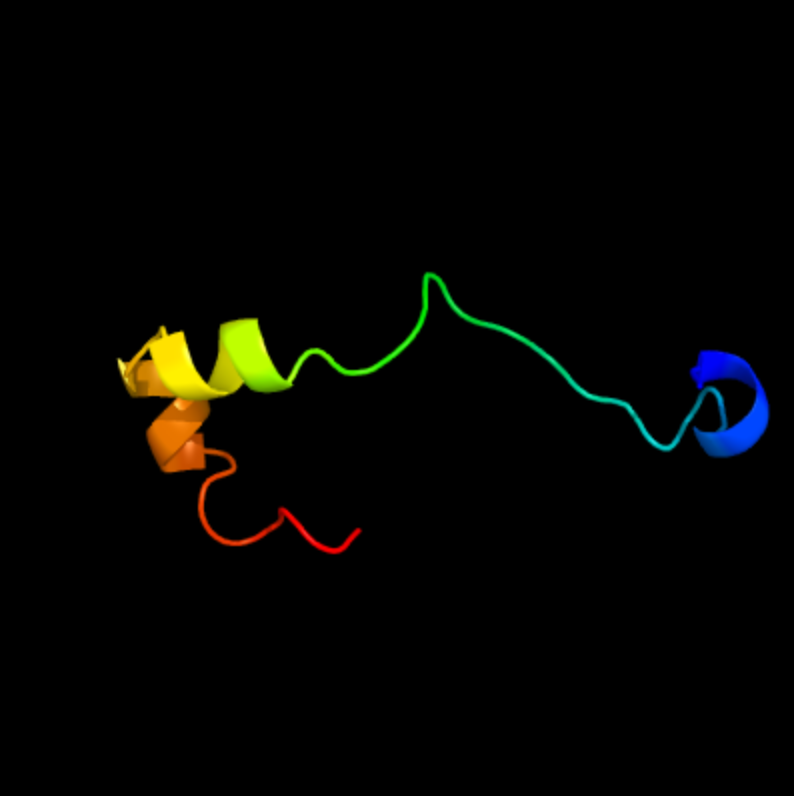


**Fig. S3.** 3D structure prediction of PA1-LRP
